# Progress in Wound-Healing Products Based on Natural Compounds, Stem Cells, and MicroRNA-Based Biopolymers in the European, USA, and Asian Markets: Opportunities, Barriers, and Regulatory Issues

**DOI:** 10.3390/polym16091280

**Published:** 2024-05-03

**Authors:** Girish K. Srivastava, Sofia Martinez-Rodriguez, Nur Izzah Md Fadilah, Daniel Looi Qi Hao, Gavin Markey, Priyank Shukla, Mh Busra Fauzi, Fivos Panetsos

**Affiliations:** 1 Departamento de Cirugía, Oftalmología, Otorrinolaringología y Fisioterapia, Facultad de Medicina, Universidad de Valladolid, 47005 Valladolid, Spain; girishkumar.srivastava@uva.es; 2 Instituto Universitario de Oftalmobiología Aplicada, Facultad de Medicina, Universidad de Valladolid, 47011 Valladolid, Spain; sofia.martinez.rodriguez22@estudiantes.uva.es; 3 Centre for Tissue Engineering and Regenerative Medicine, Faculty of Medicine, Universiti Kebangsaan Malaysia, Kuala Lumpur 56000, Malaysia; izzahfadilah@ukm.edu.my (N.I.M.F.); dr.daniellooi@cytoholdings.com (D.L.Q.H.); fauzibusra@ukm.edu.my (M.B.F.); 4 My Cytohealth Sdn. Bhd., Kuala Lumpur 56000, Malaysia; 5 Personalised Medicine Centre, School of Medicine, Ulster University, C-TRIC Building, Altnagelvin Area Hospital, Glenshane Road, Londonderry BT47 6SB, UK; markey-g2@ulster.ac.uk (G.M.); p.shukla@ulster.ac.uk (P.S.); 6 Neurocomputing and Neurorobotics Research Group, Faculty of Biology and Faculty of Optics, Universidad Complutense de Madrid, 28040 Madrid, Spain; 7 Institute for Health Research San Carlos Clinical Hospital (IdISSC), 28040 Madrid, Spain; 8 Silk Biomed SL, 28260 Madrid, Spain; 9 Bioactive Surfaces SL, 28260 Madrid, Spain; 10 Omnia Mater SL, 28009 Madrid, Spain

**Keywords:** skin injuries, therapy, caring, natural materials, regulatory issues, ISO

## Abstract

Wounds are breaks in the continuity of the skin and underlying tissues, resulting from external causes such as cuts, blows, impacts, or surgical interventions. Countless individuals suffer minor to severe injuries, with unfortunate cases even leading to death. In today’s scenario, several commercial products are available to facilitate the healing process of wounds, although chronic wounds still present more challenges than acute wounds. Nevertheless, the huge demand for wound-care products within the healthcare sector has given rise to a rapidly growing market, fostering continuous research and development endeavors for innovative wound-healing solutions. Today, there are many commercially available products including those based on natural biopolymers, stem cells, and microRNAs that promote healing from wounds. This article explores the recent breakthroughs in wound-healing products that harness the potential of natural biopolymers, stem cells, and microRNAs. A comprehensive exploration is undertaken, covering not only commercially available products but also those still in the research phase. Additionally, we provide a thorough examination of the opportunities, obstacles, and regulatory considerations influencing the potential commercialization of wound-healing products across the diverse markets of Europe, America, and Asia.

## 1. Introduction

A wound is a break of the anatomical structure of the skin that can extend further to other tissues and structures such as subcutaneous tissue, muscles, tendons, nerves, vessels, and even the bones. Skin, the largest organ by surface area in the human body, can be easily injured or burned in daily life. Cuts, scrapes, and scratches are all examples of wounds that can occur anywhere on the body, and it is essential to provide adequate care to them, to prevent infections and other complications, and to facilitate a prompt and successful recovery [1]. In this context, wound care is an essential part of the wound-healing process. The wounded person must follow several steps of wound care for absolute recovery: (1) wash and sanitize the hands to prevent infection on the wound site, (2) use clean clothes or bandages to stop the bleeding by applying the pressure at wound site, (3) clean the wound with soap or gauzes dipped in saline solution, (4) apply antiseptic solution and wound-healing ointment or products, and (5) protect the wound using sterile dressing or pad. Otherwise, a minor wound may ulcerate and create further complications and even pain, and it may require immediate consultation of a medical doctor.

**Figure 1:**
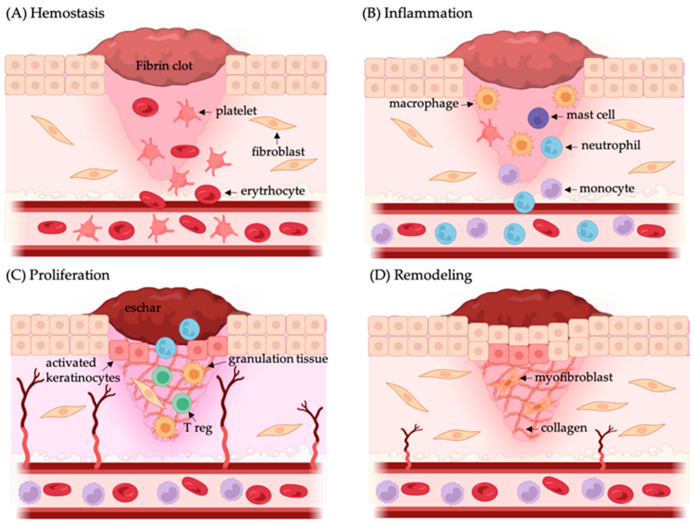
Wound-healing process. (**A**) Wound repair begins with hemostasis, where an infiltration of erythrocytes and platelets occurs and form a fibrin clot. (**B**) After that, starts the inflammation phase, where multiple immune cells reach the place of damage to control bacterial invasion. (**C**) Next, during the proliferation stage keratinocytes migrate to close the wound gap, new blood vessels are formed, and fibroblasts replace the fibrin clot with granulation tissue. (**D**) Finally, the matrix is remodeled by fibroblasts, blood vessels return and myofibroblast produce wound contraction. Image made with Biorender.

The complete wound-healing process is a tissue regeneration and growth progress at a wound spot, and it is carried out in four cascades: coagulation and hemostasis, inflammation, proliferation, and remodeling [2]. In the coagulation and hemostasis phase, the platelets get activated to form a fibrin clot immediately after the injury. The inflammation stage starts shortly after injury, it is mediated by macrophages and neutrophils to block bacterial invasion, and it is characterized by redness, pain, and heat at the wound site. During the proliferative phase, the wound bed is filled with growing cells. Growth factors and cytokines released further support the formation of new tissues and blood vessels at wound sites. Finally, remodeling is achieved by synthesizing the components of the extracellular matrix (ECM) and the subsequent transformation of granular tissue to scar tissue. Thus, wound healing is the result of interactions among ECM molecules, mediators, fibroblasts and keratinocytes, infiltrating leukocyte subtypes, cytokines, and growth factors (Figure 1) [2,3].

Depending on the duration of the healing, skin wounds can be classified into acute and chronic (as healing and nonhealing, respectively). Acute wounds progress through the above-described phases, and they exhibit well-defined signs of recovery within four weeks; on the contrary, chronic wounds do not follow the normal healing process, and they do not show obvious recovery signs in the same time interval [1]. Chronic wounds seem to be detained in one or more phases of the wound-healing process due to influence of already activated pathophysiological mechanisms, as for example, the pathologic processes in the diabetic patients, which make wound recovery require much more time. The antiseptic characteristics and wound-healing promoters, with their action potentials, counteract these complications. They regulate hemostasis and inflammation at the wounded site, promote cell proliferation, and facilitate tissue remodeling, ultimately leading to wound healing and recovery. Nevertheless, the healing process of a wound can be complicated by several factors, including age, health conditions, chronic conditions, and genetic heritage, which can hinder the process, and impede overall recovery. Similarly, sick people would take longer to heal and recover than healthy ones. On the other side, chronic wounds are more difficult to treat and place a significant and often underestimated burden on the individual, on the healthcare systems, and on the society as a whole, since individuals with chronic wounds face more challenges than those with acute ones [4,5]. Furthermore, there are wounds that do not heal properly, e.g., those due to accidents or exposure to chemicals. This picture is likely to be made worse by lifestyle, which silently causes diabetic wounds, pressure sores (bedsores), leg and foot ulcers, and ulcers related to venous or arterial disease.

In addition to the above considerations, it is crucial to note that wound healing is negatively correlated with age [4,5] and, as the global population continues to age, this offers a substantial market opportunity.

According to the United Nations (UN), in 2019 there were 703 million people in the world aged ≥ 65 [6], and this number is projected to double to 1.5 billion by 2050 [6]. In early 2018 there were 101.1 million older people aged ≥ 65 living in the EU countries, which is almost one fifth (19.7%) of the total population. During the next three decades, this figure is projected to rise to 149.2 million inhabitants in 2050 (28.5% of the total population) [7]. In countries like the UK, according to the Office of National Statistics (ONS), in 2018 there were nearly 12 million people aged ≥ 65, and this number is expected to be over 20.6 million by 2048 [8]. In Spain, according to the Spanish National Statistics Office (INE) and the UN, in 2019 there were 9 million people aged ≥ 65, and their projections suggest that by 2050 more than 30% of the Spanish population (almost 13 million people) will be ≥65 years old, making Spain one of the world’s top 10 countries in terms of aging population [6]. According to the US Census Bureau, the number of Americans ages ≥ 65 is projected to nearly double from 52 million in 2018 to 95 million by 2060, and the ≥65 age group’s share of the total population will rise from 16.0% to 23.0% [9]. According to the US Census Bureau, in Asia, the number of older people is expected to nearly triple over the next four decades. There were an estimated 414 million Asians aged ≥ 65 in 2020, projected to grow to more than 1.2 billion in 2060, which implies that one out of every 10 people in the world will be an Asian [10]. Most of the older population in Asia will be residing in the Eastern subregion (491 million) and the Southern subregion (464 million), where China and India are located, respectively. Among Asian subregions, 33.7% of Eastern Asia’s population is projected to be age ≥65 by 2060. In contrast, Southern Asia (18.6%) and Western Asia (17.9%) are expected to have the lowest proportions of older people of their regional total populations [10]. In Asian countries, Malaysia’s ageing population is growing at a faster-than-expected rate where, according to the Department of Statistics of Malaysia, more than 15% of its population will be ≥65 by 2050 [11].

The dramatic increase in the elderly population will pose a major challenge to the healthcare systems due to the expensive nature of wound management. The rising number of surgical cases and the increasing prevalence of chronic diseases worldwide have generated a significant global demand for wound-healing products. This demand is further amplified by the ongoing demographic shift towards an ageing population, resulting in a steady increase in the demand for such products, thereby presenting a challenge for health care systems worldwide. 

In Europe and the USA, the healthcare sector already has a significant demand for wound-healing products, thus playing a key role in the healthcare market, making them the largest wound-healing product markets worldwide. The prevalence and economic burden of wounds are significant. US alone spends over $25 billion annually, to treat chronic wounds in over 6 million patients [12]. In the UK, the annual NHS cost of wound management is £8.3 billion, of which £2.7 billion and £5.6 billion are associated with managing healed and unhealed wounds, respectively [13]. In Europe, 2–4% of the total health annual expenditure is used for wound management [14]. In developing countries like India, approximately 3–4% of all diabetic population have a foot problem and consume 12–15% of the healthcare resources [15], while the Malaysian Ministry of Health’s budget for wound care reaches $5 million per year [16] and the other Southeast Asian countries are in a similar situation.

**Figure 2:**
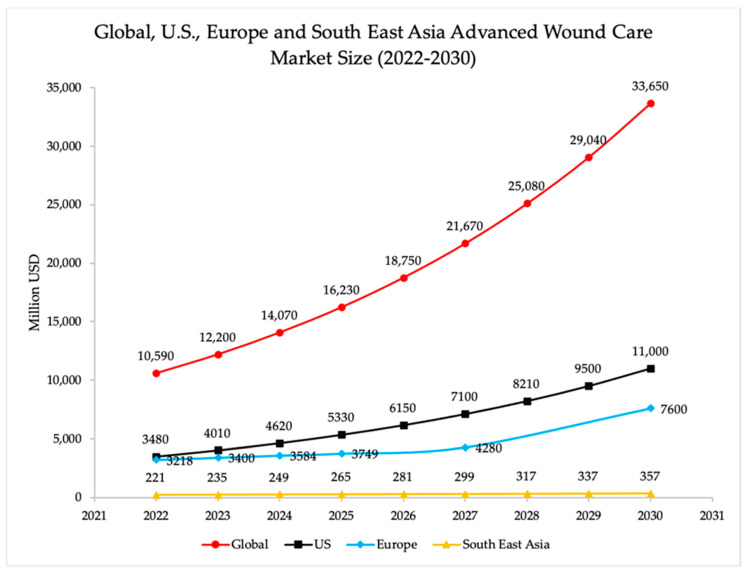
Global, US, Europe and South East Asia advanced wound care market sizes between 2022 and 2030. For the global market, a mean annual increase of 3474 million USD is foreseen, while for the US market, a mean annual increase of 1130 million USD is estimated. For the European market, a mean annual increase of 2482 million USD is foreseen, while for the Southeast Asian market, a mean annual increase of 17 million USD is estimated.

Several reports are published by the market researchers showing past, current, and predicted global and regional wound care market size. Some of them are published with the title ‘Wound Care Market Size,’ others with ‘Advanced Wound Care Market Size,’ and a few focus on specific wound treatments such as anti-infective therapies and biologics. It must be clarified that advanced wound care strategies are applied with patients when standard wound care has failed to treat wound. Between 2022 and 2030, analysts estimate a total increase of 23,060 million USD, with a mean annual increase of 3474 million USD for the worldwide market, and a total increase of 7520 million USD, with a mean annual increase of 1130 million USD for the US market. In the same period, for the Europe market they estimate a total increase of 4382 million USD, with a mean annual increase of 2482 million USD, and a total increase of 136 million USD for the Southeast Asia market, with a mean annual increase of 17 million USD (Figure 2) [17,18,19].

**Figure 3:**
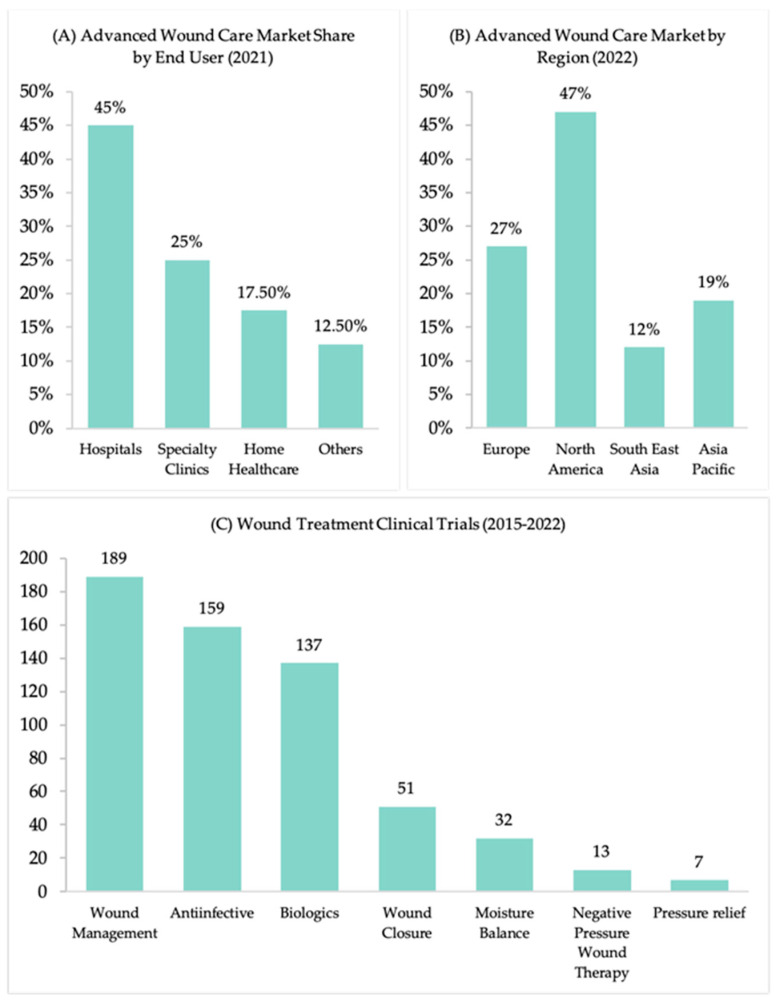
(**A**) Advanced wound care share by end user (2021 data). The hospital sector commanded the market with the highest revenue share, followed by specialized clinics, home care, and finally, other segments. (**B**) The leading market for wound healing globally was North America, trailed by Europe, followed by the Asia-Pacific region, and lastly, South East Asia (2022 data). (**C**) Recent clinical trials on wound treatment classified into various categories, including wound management, anti-infective therapies, biologics, wound closure techniques, moisture balance strategies, negative pressure wound therapy, and pressure relief methods. The numbers represent the count of interventional clinical trials conducted between 2015 and 2022, including those that are recruiting, not yet recruiting, actively recruiting, completed, or enrolling by invitation.

Furthermore, the data identifying the leading markets for the advanced wound care market confirm that North America, covering 47% of the market, leads globally. It is followed by Europe, the Asia-Pacific region, and, lastly, South East Asia (Figure 3) [17,20]. In this market size, the hospital sector commanded the market with the highest revenue share, followed by specialized clinics, home care, and finally, other segments (Figure 3) [17]. The growing demand from the health sector and its vast market are driving research and development efforts to create new wound-healing products. Consequently, there are currently several clinical trials underway for wound treatment. These trials are categorized into different types, which are summarized in Figure 3 [21].

Moreover, the chronic disease epidemic has significantly impacted the healthcare landscape, with conditions like diabetes becoming increasingly prevalent (around 15% and 25% of patients with diabetes may develop a diabetic foot ulcer during their lifetime) [22]. This surge in chronic diseases has consequently led to a higher incidence of wounds, necessitating advanced and targeted healing interventions. As individuals grapple with these health challenges, there is a growing awareness among both patients and healthcare professionals about the advantages offered by novel therapies. This heightened awareness has fostered a preference for treatments that promise improved outcomes and faster recovery. Recognizing the urgent need for effective solutions, the healthcare market has identified substantial potential in the development and commercialization of advanced wound-healing products. This recognition has spurred considerable investments in research and development, driving innovation in the field. The market’s enthusiasm to address the challenges posed by chronic diseases and their associated wounds is evident in the increased focus on creating advanced and specialized therapeutic options.

Below we present the recent breakthroughs in natural wound-healing products, incorporating biopolymers, stem cells, and miRNA, encompassing both research and commercial domains. Additionally, we present a comprehensive overview of the regulatory landscape in the European, American, and Southeast Asian markets, pivotal for the successful commercialization of these innovations. These advancements provide a synergistic approach, elevating the effectiveness and precision of wound healing while minimizing potential side effects. Achieving global market success hinges on harmonizing regulatory frameworks and establishing evidence-based approvals. In essence, the integration of these pioneering components marks a significant frontier in wound care, with its realization contingent upon overcoming challenges and fostering collaboration across European, USA, and Asian markets. 

In the existing literature, one can scarcely find an article that comprehensively discusses recent research perspectives and market values concerning natural compounds, stem cells, and miRNAs-based polymers for wound healing. Additionally, this paper delves into the opportunities, barriers, and regulatory issues across European, USA, and Asian markets. This comprehensive approach brings and retains the attention of readers. Thus, this distinctive focus sets this article apart from the current literature, rendering it particularly intriguing for researchers, regulators, and marketers alike.

## 2. Wound-Healing Biopolymers, Based on Natural Compounds

### 2.1. Natural Compound-Based Regenerative Biopolymers for Wound Healing in Research

Since the process of wound healing is dynamic and highly complex, effective management of wounds at the initial stages is the best possible prevention strategy. Therefore, the development of therapeutics by using bioactive materials has attracted the interest of both scientific and industrial communities; the scientific wound community, because of the possibilities they offer to interact and modulate the healing biomolecular processes, and industry, because of the promising outcomes for fabricating smart wound care dressings. Thanks to the technological advances in bioengineering, nanotechnology, materials sciences, and regenerative medicine, we can create functional biomaterials with improved physical–chemical, and structural characteristics, to meet the highest requirements and quality standards of current wound care, as, for example, to achieve restoration of lost tissue integrity and scarless healing [23]. 

The incorporation of biopolymers and recent advances in the medical, pharmaceutical, and bioengineering fields helps to develop new strategies for the treatment of wounds, and, in particular, for chronic nonhealing ones. Indeed, wound-healing products are being fabricated from different polymer biomaterials, incorporating various bioengineering techniques, for example, films, hydrogels, foams, and sponges, some of which incorporate bioactive agents to enhance their healing properties. 

Many of the today’s biopolymers could enhance performance in wound healing and mimicking ECM [24]. They are mainly extracted from their natural origin, including animals (chitosan, collagen, hyaluronic acid), plants (cellulose, starch, rubber), bacteria (exopolysaccharides, bacterial cellulose), fungi (chitin), and algae (alginate). These biopolymers are safe to be used for skin regeneration because of their excellent properties, such as biodegradability, biocompatibility, lower antigenicity, and similarity to macromolecules recognized by the human body [25]. Prior to achieving a key role in the wound-healing process, biopolymers offer multiple benefits with their properties including anti-inflammatory, antioxidants, antibacterial, or other target actions to enhance the regeneration process (Table 1). 

The review paper by Yang et al. covers polysaccharides as ideal materials for self-healing hydrogels [32]. The authors also discussed the derivatives of cellulose, alginate, hyaluronic acid, and chitosan together with their preparation methods. Following that, collagen is also one of the natural biopolymers commonly used as a biomaterial for tissue engineering applications. It is the human body’s most abundant protein and can be easily manufactured in different forms [33]. Another article discussed the ability of collagen to be fabricated into three types of 3D scaffolds, in the form of hydrogel, sponge, and film by using collagen extracted from the ovine tendon. All the collagen scaffolds demonstrated significantly higher attachment and were biocompatible with the human dermal fibroblast cells [34]. In contrast to the other biopolymers mentioned above, silk fibroin has become remarkable as a natural biopolymer often used for biomedical applications, including wound healing [35]. In a research article published in 2021, Dong et al. observed that silk fibroin injectable hydrogel can enhance wound-healing efficiency in burn wounds [36]. The authors incorporated ciprofloxacin into graphene oxide/silk fibroin injectable hydrogel as a multifunctional wound dressing to provide effective antibacterial, cell compatible, and in vivo wound closure actions. In addition, another notable biopolymer with therapeutics applications is carrageenan. It is extracted from several red seaweed species, mostly from members of the Rhodophyceae class, such as *Chondrus crispus*, *Eucheuma cottonii*, *Eucheuma spinosum*, and *Gigartina stellate*, having a hydrogalactose and galactose units linked by glycosidic bonds. Neamtu et al. [37] mentioned in their review paper that carrageenan has low cytotoxicity, antimicrobial, and antioxidant qualities, thus it does not stick to the wound bed. This biopolymers’ versatility in formulations and applications makes them a candidate for developing a novel wound-healing product.

### 2.2. Natural Compound-Based Regenerative Biopolymers for Wound Healing in the Market

A high number of wound-healing products developed using biopolymers have been successfully launched in the market. A list of recently developed and commercially available products is detailed below (Table 2). Key information on each biopolymer, including structural components, strengths, limitations, and commercial information, is also included. Most of them have shown good market growth due to their beneficial characteristics to patients. Nevertheless, the limitations and deficiencies including the health problems from their use cannot be avoided. The Algisite M Calcium Alginate Dressing is based on Alginate Calcium which allows many benefits to patients for wound healing; however, on its use it is noted that Ca^2+^ divalent cations can be released and exchange with other monovalent cations in the surrounding media, resulting in the dissolving of the alginate gel [38]. The collagen-based Suprasorb C Collagen Wound Dressing has shown low mechanical strength and low antiseptic properties with several beneficial properties for patients [39]. Any health problem with the product Kito Activator Chitosan Wound Healing Hydrogel Barrier has not been identified till now, nevertheless it is a Chitosan Hydrogel [40]. The Medihoney Honeycolloid Leptospermum Hydrocolloid Dressings contain active leptospermum honey and hydrocolloidal gelling agent, but it may increase the level of exudate upon initial use. It is only suitable to treat moderately exuding wounds [41]. The Hyperoil that contains Neem (Azadirachtin) and Hypericum (Hyperforin), and the combined overall effect is available in both gel and oil formulation; however, it showed low antimicrobial properties for wound healing [42]. The Fibracol Plus Collagen Wound Dressing with Alginate is fabricated using 90% collagen and 10% calcium alginate but, in this case, the Ca^2+^ divalent cations can be released and exchange with other monovalent cations in the surrounding media, resulting in the dissolving of the alginate gel as has been seen in the case of the Algisite M Calcium Alginate Dressing [43]. All these deficiencies support the continuity of investigation for finding and developing better alternatives.

Technological advancements in bioengineering, nanotechnology, materials sciences, and regenerative medicine enable the creation of functional biomaterials with improved characteristics to meet the highest quality standards of current wound care. The incorporation of recent advances in medical, pharmaceutical, and bioengineering fields into biopolymers helps develop new strategies for treating wounds, especially chronic nonhealing ones. Various types of biopolymers derived from natural sources are considered safe for skin regeneration due to their excellent properties such as biodegradability, biocompatibility, and similarity to human body macromolecules. Biopolymers offer multiple benefits including anti-inflammatory, antioxidant, and antibacterial properties, and numerous wound-healing products developed with biopolymers have been successful, particularly those based on natural compounds, showing good market growth due to their beneficial characteristics for patients.

## 3. Stem Cell-Based Wound-Healing Biopolymers

### 3.1. Stem Cell-Based Regenerative Biopolymers for Wound Healing in Research

Stem cells, particularly mesenchymal stem cells (MSCs), hold enormous potential for accelerating tissue restoration and wound healing through their immune-modulating, regenerative, and paracrine properties. MSCs secrete bioactive molecules like cytokines, growth factors, and neurotrophic factors that promote tissue regeneration and exhibit anti-inflammatory, angiogenic, and immunomodulatory effects. They can be administered directly or via their secretome, which can be obtained and used as a safer alternative [64,65,66,67,68]. Induced Pluripotent Stem Cells (iPSC) are the newest class of stem cells with the potential and limitations to achieve wound healing [69]. However, plant stem cells have distinct characteristics compared to human stem cells, but they can still benefit wound healing. Although the growth factors and proteins produced by plant stem cells do not have the same effects in humans as they do in plants, derivatives of plant stem cells can stimulate the production of human skin cells and collagen, while providing beneficial nutrients to the skin. Additionally, extracts from plant stem cells possess antioxidant, antibiotic, and anti-inflammatory properties that support and enhance the wound-healing process. While plant stem cells cannot directly repair and regenerate human skin tissue as they would in plants, they offer valuable contributions to wound healing through their unique properties [70]. 

In the laboratory, researchers are exploring the use of stem cell-based wound-healing biopolymers as a potential approach to enhance the healing process of wounds. These biopolymers, which are natural or synthetic materials, are engineered to support the growth, differentiation, and function of stem cells specifically for wound-healing applications. Stem cell-based wound-healing biopolymers can be designed to provide a three-dimensional structure that mimics the ECM, creating an environment conducive to stem cell attachment, proliferation, and differentiation. These biopolymers can be modified to have specific physical and chemical properties, such as biodegradability, mechanical strength, and surface characteristics, to optimize their interaction with stem cells and promote wound healing. One approach involves incorporating stem cells directly into the biopolymer matrix. This can be achieved by encapsulating stem cells within hydrogel-based biopolymers or seeding stem cells onto porous scaffolds. The biopolymer matrix provides structural support and acts as a delivery system for bioactive factors secreted by the stem cells. These factors, including growth factors and cytokines, can stimulate various cellular processes involved in wound healing, such as cell proliferation, angiogenesis, and ECM remodeling. Another approach focuses on utilizing the secretome of stem cells. The secretome refers to the complex mixture of bioactive molecules, including growth factors, exosomes, and other signaling molecules, which are released by stem cells. Biopolymers can be engineered to capture and release these bioactive molecules, either by direct incorporation or through surface modifications. By presenting these signaling factors in a controlled manner, the biopolymer can promote wound healing by modulating cellular activities and promoting tissue regeneration. In this context, researchers are investigating different combinations of stem cells and biopolymers, as well as optimizing their formulation and delivery methods. They are studying the biocompatibility, mechanical properties, degradation kinetics, and release profiles of these biopolymers to ensure their safety and efficacy for wound-healing applications. 

Furthermore, researchers are exploring the use of advanced techniques such as 3D bioprinting and microfabrication to precisely engineer complex biopolymer structures and create customized wound-healing platforms [71,72,73,74]. Catanzano et al. reviewed the design, characterization, and evaluation of wound-healing products integrated with growth factors [75]. Some criteria should be considered when growth factors are loaded into the carrier system (biopolymer), such as their encapsulation efficiency, stability, and controlled release into the wound setting. However, the high cost is one of the limitations when considering growth factor enrichment. Furthermore, Mashiko et al., have found that incorporating adipose-derived stem cells into a recombinant collagen scaffold demonstrated superior wound-healing progress compared to the recombinant protein scaffold alone [76]. The structural arrangement of the skin layer is similar to normal skin after treatment with biopolymers and biomaterials [77]. Therefore, good healing potential and results were obtained using these platforms of topical wound-healing products, so-called bioactive dressings, which can be considered the best treatment for repairing full-thickness wounds and provide benefits for patients in the future. Thus, stem cell-based wound-healing biopolymers in the lab represent a promising area of research, aiming to harness the regenerative potential of stem cells and the supportive properties of biopolymers to improve wound-healing outcomes. Further studies and advancements in this field hold the potential for the development of innovative therapies that could revolutionize the treatment of chronic wounds.

### 3.2. Stem Cell-Based Regenerative Biopolymers for Wound Healing in the Market

Biopolymers provide a favorable microenvironment for stem cells to proliferate, differentiate, and accelerate wound-healing processes. In recent years, there has been noteworthy progress in the development and commercialization of these innovative biopolymers, leading to their availability in the market. As mentioned previously, one of the key advantages of stem cell-based wound-healing biopolymers is their ability to enhance tissue regeneration through the release of bioactive factors by the embedded stem cells. These factors promote angiogenesis, collagen synthesis, and recruitment of endogenous cells, which collectively contribute to wound closure and tissue repair. Additionally, these biopolymers offer advantages such as biocompatibility, biodegradability, and ease of application, making them suitable for a wide range of wound types and sizes. Furthermore, the commercial availability of these products allows clinicians to access standardized and quality-controlled formulations, ensuring consistent and reproducible outcomes. There are several components related to wound-healing stem cells that are available in the market for clinical use. These components include MSCs, growth factors, extracellular vesicles and exosomes, scaffold materials, and hydrogels and dressings. 

As described previously, MSCs is the most commonly used type of stem cells for wound healing because they possess the ability to differentiate into different cell types involved in wound healing and secrete bioactive factors that promote tissue regeneration. Various growth factors derived from stem cells are available in the market for wound-healing applications. These growth factors include epidermal growth factor (EGF), platelet-derived growth factor (PDGF), vascular endothelial growth factor (VEGF), and transforming growth factor-beta (TGF-β) [78]. These factors play a crucial role in stimulating cell proliferation, angiogenesis, and collagen synthesis, leading to accelerated wound healing. Stem cells release extracellular vesicles, including exosomes, which contain a variety of bioactive molecules such as proteins, lipids, and nucleic acids. These extracellular vesicles have shown promising therapeutic effects in wound healing by promoting cell migration, angiogenesis, and tissue regeneration [79]. Commercially available exosome-based products are emerging as potential treatments for wound healing. Stem cell-based wound healing often involves the use of biocompatible scaffold materials that provide a three-dimensional structure for stem cell attachment, proliferation, and differentiation. These scaffolds can be made from natural or synthetic materials and are designed to mimic the ECM. They provide a supportive environment for stem cells to enhance wound-healing processes. Stem cell-based wound-healing hydrogels and dressings are also available in the market. These products are designed to provide a moist environment for wound healing and can be loaded with stem cells or stem cell-derived factors. Hydrogels and dressings help maintain optimal conditions for stem cell activity, protect the wound from infection, and facilitate the healing process. Thus, these components represent the diverse range of products available in the market that harness the potential of stem cells for wound healing. They offer a variety of approaches to promote tissue regeneration, angiogenesis, and accelerated wound healing, providing clinicians with valuable tools in the treatment of chronic wounds. 

Table 3 presents a compilation of novel stem cell-based wound-healing products developed in recent years. Among them, allo-APZ2 comprises ABCB5-MSCs, TruStem incorporates hematopoietic and MSCs, and XSTEM utilizes human stem cells along with Integrin α10β1 [80,81,82,83]. The first and last products mentioned are currently undergoing clinical trials. TruStem demonstrates an extended response time, taking weeks to months to observe therapeutic effects. Additionally, there are many other cell-based, growth factor-based, and natural ECM-based wound-healing products available in the market. A few to mention are: ReGenerCell™, DermaPure^®^, Grafix^®^, NuCel^®^, Myskin^®^, Cryoskin, ReCell^®^, BioDfentor, BioDfence^®^, and Appligraf^®^. ReGenerCell™ utilizes a patient’s own skin cells, which are processed and sprayed onto wounds using the ReCell^®^ device (Avita Medical, Inc., Valencia, CA, USA). The product aims to promote wound healing and reduce scarring by delivering a population of cells, including keratinocytes, to the wound site [84]. DermaPure^®^ is an autologous (patient-derived) cell therapy that utilizes a patient’s own cells to create a bioactive wound dressing. The product is designed to enhance wound healing by providing a cellular and growth factor-rich environment [85]. Grafix^®^ is a cryopreserved placental membrane-based product that contains various components, including MSCs, growth factors, and ECM proteins. The product aims to facilitate wound healing by providing a regenerative environment [86]. Therefore, technically sound approaches that facilitate wound healing are found at the core of all these wound-healing products. It is also noted that many of them have been using the amniotic membrane as an ECM to support and retain the cells and growth factors. The amniotic membrane’s natural ECM properties, including its protective barrier function, abundance of bioactive factors, anti-inflammatory effects, and support for tissue regeneration, makes it a highly favorable choice for promoting effective wound healing and achieving optimal clinical outcomes [87]. However, several of these products share common limitations, including possibility of contaminations, compatibility issues with allogeneic cell source, and relatively modest wound-healing outcomes. Moreover, wound-healing products need to undergo rigorous regulatory scrutiny to ensure the safe and ethical use of stem cells derived from both human and animal sources in clinical settings.

MSCs, the most commonly used type of stem cells for wound-healing, growth-factor-derived, such as EGF, PDGF, VEGF, and TGF-β, or extracellular vesicles like exosomes show therapeutic effects in wound healing by promoting cell migration, angiogenesis, and tissue regeneration. Advanced techniques like 3D bioprinting and microfabrication are being utilized to engineer complex biopolymer structures tailored for wound-healing applications. Studies have shown promising results with stem cell-based biopolymers, demonstrating improved wound-healing outcomes compared to conventional treatments. However, further research is needed to fully exploit their potential and revolutionize the treatment of chronic wounds and challenges, such as the high cost of growth factor enrichment and optimization of growth factor delivery systems, remain.

Commercially available products employing stem cells or stem cell-derived factors for wound healing include hydrogels, dressings, and scaffold materials providing a supportive environment for stem cell activity, as well as products, like ReGenerCell™, DermaPure^®^, and Grafix^®^, employing patient-derived cells or placental membrane-based components to enhance wound healing, offer very promising outcomes and could become the leaders of the market if they will be able to overcome their actual limitations, such as contamination risks and compatibility issues with allogeneic cell sources. Additionally, stringent regulatory scrutiny is essential to ensure the safe and ethical use of stem cell-derived products in clinical settings.

## 4. MicroRNA-Based Wound-Healing Biopolymers

### 4.1. MicroRNA-Based Regenerative Biopolymers for Wound Healing in Research

MiRNAs are small noncoding RNAs that average 22 nucleotides in length. Primarily transcribed from DNA sequences, they are known to play a key role in the regulation of gene expression. Mature miRNAs predominantly interact with the 3′ untranslated region (3′UTR) of messenger RNA (mRNA) resulting in the degradation and translational repression of miRNA transcripts. However, they are also known to interact with other regions including the 5′UTR, gene promoter regions and coding sequences [88]. There are currently over 2500 known miRNAs in humans, some of which target mRNAs involved in a wide range of biological and pathophysiological processes [89]. Due to this, it is vital to consider not only how the presence of an individual miRNA will benefit in wound healing, but also how it may impact gene expression in surrounding tissue [90].

As previously stated, wound healing is a multistep process involving coagulation and hemostasis, inflammation, proliferation, and remodeling of tissue. Following tissue damage, miRNAs are transiently expressed to suppress excessive inflammatory responses, promote proliferation and migration of keratinocytes, and improve collagen expression and the ECM remodeling [89]. In recent years, many miRNAs have been discovered that contribute to wound healing during inflammation (21, 125b, 132, 146a, 155, 223), proliferation (21, 31, 99, 132, 210), and remodeling (29a, 29b, 29c, 192) phases, detailed in Table 4 [89]. In order to exploit these miRNAs to enhance wound healing using biopolymers several criteria must be met; (1) the biopolymer must be positively charged to facilitate adhesion as all miRNAs are negatively charged, (2) cellular uptake of miRNAs at the site of the wound must be stable, avoid lysosomal degradation, and positively contribute to the wound-healing process, (3) the biopolymer/miRNA complex must be nontoxic and biocompatible with the surrounding tissue and microenvironment [91,92]. Biopolymers can also overcome limitations of traditional viral vector-based delivery systems, such as limited capacity for genetic cargo resulting in poor immunogenicity, possibility of viral mutagenesis leading to malignant transformation of cells, and other unwanted cytotoxic effects [93].

Wound treatment strategies using miRNAs predominantly focus on either upregulating miRNAs that have a positive effect on wound healing or downregulating those which have a negative effect. This task is complicated by changes in miRNA expression throughout the wound-healing process, alongside differences in miRNA expression profiles in different tissue types and in those with chronic wounds such as venous ulcers (VUs) and diabetic foot ulcers (DFUs) [94]. Due to this, many natural biopolymer-based products incorporating miRNAs often focus on treating acute cutaneous wounds (burns, bacterial infection) or chronic wounds (VUs, DFUs) that are associated with unique miRNA profiles. This approach has been successful in recent in vivo studies in mice for both acute and chronic wounds. Using a Chitosan, Puerarin hydrogel (C@P) Zeng et al. [95] successfully inhibited miR-29ab1, resulting in the downregulation of IL-1β and TNF-α secreted by M1 macrophages, significantly accelerating the wound-healing process in diabetic mice. Similarly, Zhang et al. [96] used a Jiang Tang Xiao Ke (JTXK)-based hydrogel to inhibit miR-139-5p to promote acceleration of healing process in *S. aureus* infected wounds.

Another recent study comparing miRNA expression in chronic venous insufficiency patients against a healthy population of similar age resulted in the identification of seventeen pathologically relevant miRNAs that contributed to persistent inflammation and proliferation phase inhibition. The study found that three miRNAs (34a, 424, 516) were consistently upregulated in patients with VUs, the most common chronic nonhealing wound type [97]. Similarly, patients suffering from diabetes mellitus complicated by chronic nonhealing wounds such as DFUs exhibit an overexpression of miR-29ab1 from M1 macrophages. All this research confirms sufficient progress involving miRNA-based wound-healing biopolymers in recent years.

**Table 4:** miRNAs associated with the wound-healing process. Wound healing phase and associated wound type have been listed alongside a brief synopsis of the miRNA function that promotes wound healing.

Phase	miRNA	Function	Associated Wound Type	Reference
Inflammation	miR-23b	Reduces pro-inflammatory cytokines	Acute	[98]
Inflammation	miR-27b	Downregulation of miR-27b promotes fibroblast proliferation	Acute (burn)	[99]
Inflammation	miR-34a/b	Promotes production of chemokines and cytokines prolonging inflammation	Chronic (venous ulcers)	[100]
Inflammation	miR-146a	miR-146a deficiency is associated with enhanced inflammatory response in diabetic wounds	Diabetic foot ulcers	[101]
Inflammation	miR-203	Inhibits proliferation and migration of keratinocytes	Diabetic foot ulcers	[102]
Inflammation	miR-223	Enhances clearance of *S. aureus* through neutrophil activation	Acute (Bacterial infection)	[103]
Inflammation/Proliferation	miR-21	Down-regulates PTEN/RECK and activates MAPK/ERK cascade, inhibiting inflammation	Acute	[104]
Inflammation/Proliferation	miR-31	Enhances keratinocyte proliferation and migration	Acute	[105]
Inflammation/Proliferation	miR-125b	Interacts with TP53INP1 promoting cell migration and proliferation	Acute	[106]
Inflammation/Proliferation	miR-132	Promotes endothelial cell proliferation, migration, and angiogenesis	Acute (burn)	[94]
Inflammation/Proliferation	miR-139-5p	Suppresses miR-139-5p expression enhancing neutrophil migration and proliferation in *S. aureus* wounds	Acute (Bacterial infection)	[96]
Inflammation/Proliferation	miR-126	Promotes endothelial cell proliferation, migration, angiogenesis and inhibits apoptosis	Acute (burn)	[107]
Inflammation/Proliferation	miR-155	Promotes keratinocyte migration and cellular proliferation	Acute	[108]
Proliferation	miR-99a/b	Suppresses keratinocyte migration and cellular proliferation	Acute (slow healing)	[109]
Remodeling	miR-29a/b/c	Represses extracellular matrix expression and fibroplasia, preventing fibrotic scars	Acute (scar prevention)	[110]
Remodeling	miR-192	Enhances collagen expression targeting SMAD-interacting protein 1 (SIP1)	Acute	[111]

### 4.2. MicroRNA-Based Regenerative Biopolymers for Wound Healing in the Market

Research into small RNA-based therapeutics containing miRNAs or small interfering RNAs (siRNAs) is currently in a state of relative infancy, and although there are no commercially available miRNA-based therapeutics today, over 2000 patents have been filed relating to their use, with several studies having reached the latter stages, i.e., Stages III and IV, of clinical trials [112]. There are, however, numerous obstacles that hinder progress through clinical trials, such as wound healing being a species-specific process, meaning no animal model can fully predict clinical outcomes in humans. There is also the potential for unwanted off-target effects as many miRNAs can upregulate/repress numerous genes. This coupled with a limited availability of positively charged, affordable biopolymers, capable of carrying sufficient genetic cargo that can survive rapid degradation of miRNAs is another key factor. These obstacles, coupled with the robust regulations surrounding gene-based therapeutics, are pivotal to understanding why miRNA-based biopolymers have yet to become commercially available despite several products having had recent success in clinical trials [113,114]. 

Currently, treatment strategies for chronic and slow healing wounds (severe burns, VUs, DFUs) often require lengthy hospitalization, constant reapplication of surgical dressings at the site of the wound, courses of nonsteroidal anti-inflammatory drugs (NSAIDs), and topical antimicrobial ointments. Cellular/tissue-based products capable of accelerating the wound-healing process are often costly and usually only administered after 4 weeks of standard care [115]. With the global financial burden of wound treatment estimated between $28.1–$96.8 billion, of which chronic wounds represent a huge portion, identification of wound specific miRNAs is vital to developing biopolymer-based miRNA treatment strategies. Chronic wounds are also more common in the elderly and as global life expectancy and healthcare costs continue to rise so too will this financial burden [18].

There has been significant progress in miRNA-based wound-healing biopolymers in recent years. Natural biopolymer-based products incorporating miRNA, either to upregulate miRNAs with positive effects on wound healing or to downregulate those with negative effects tested in acute and chronic wounds, were successful in both cases, as deduced by the over 2000 patents in clinical trials, still no products are commercially available for miRNA-based therapeutics. However, there are significant obstacles impeding progress, including the species-specific nature of wound healing, potential off-target effects of miRNAs, and limited availability of suitable biopolymers for delivering genetic cargo. These challenges, along with stringent regulations surrounding gene-based therapeutics, explain why miRNA-based biopolymers have not yet become commercially available despite promising clinical trial results. Furthermore, current treatment strategies for chronic and slow-healing wounds often involve lengthy hospitalization, frequent application of surgical dressings, NSAIDs, and topical antimicrobial ointments. Cellular and tissue-based products that accelerate wound healing are costly and typically administered after several weeks of standard care. With chronic wounds representing a significant portion of the global financial burden of wound treatment, identifying wound-specific miRNAs is crucial for developing effective biopolymer-based miRNA treatment strategies, especially considering the increasing prevalence of chronic wounds in the elderly population and rising healthcare costs globally.

## 5. Market Growth of Wound-Healing Products

### 5.1. USA, Europe, and Asia’s Potential to Meet the Growing Demand for Biomaterials

The demand for biomaterials is expected to grow in the coming decade. Based on a recent survey, the global market value for biomaterials was estimated at $60–$100 billion in 2020, despite the economic downfall introduced by the SARS-CoV-2 (COVID-19) pandemic. The latest market survey and analysis (2020 to 2030) predicts that the market value of biomaterials will triple at a compound annual growth rate (CAGR) of ≥12% to reach ≥$200 billion (USD) by the end of the decade (Table 5) [116,117]. Market surveys were segmented according to commonly referenced biomaterial products, such as medical implants, diagnostic tools, tissue engineering, and regenerative medicine or drug delivery tools. 

Evidently, the USA has played a leading role in driving innovations, applications, and information technology in the introduction of biomaterials into the wound care market. As global pioneers, the strength borne by USA is in the presence of large market players [118]. The availability of investors for research and development, and the healthcare infrastructures to support such efforts are never scarce. Some of the dominant conglomerates, not confined by geographical factors, were incidentally based in the USA. These include 3M Healthcare, Stryker, and Johnson & Johnson [116,117,118,119,120,121]. Another prominent factor from the USA belongs to their impactful lab-to-market transitions. Solely having investment power is not definitive enough to determine success but conducting thorough market analysis is within their specialty. This process is crucial in securing credibility, resourcefulness, and predicting the future of any product or service. It compartmentalizes all the necessary information including supply and demand, client preferences, competitors, and other market-related variables. The presence of major regulatory bodies originating from the USA like the Food and Drug Administration (FDA) quickens decision-making processes for researchers and scientists to channel their products into the market. 

Europe, similar to the USA, stands at the forefront of pioneering innovative biomaterials for wound-healing applications, driven by its robust scientific research capabilities and extensive network of universities, research institutions, and industry collaborations. What sets Europe apart is its strong emphasis on translational research, enabling a seamless transition from laboratory discoveries to practical clinical applications. This is achieved through close partnerships between academic researchers, industry stakeholders, and healthcare professionals, facilitating the integration of scientific advancements into real-world patient care. To ensure the safety and effectiveness of these wound-healing products, the European Medicines Agency (EMA) and national regulatory bodies uphold rigorous testing and evaluation procedures, assessing parameters such as biocompatibility, stability, and performance [122]. Compliance with these stringent regulatory requirements guarantees that biomaterial-based wound-healing products meet elevated standards of quality, safety, and efficacy prior to their approval for clinical use [122]. By fostering ongoing research, encouraging innovation, and promoting collaboration, Europe is poised to effectively meet the growing demand for advanced biomaterials, making significant contributions towards enhancing wound-healing outcomes [123].

Asia possesses immense potential to meet the escalating demand for biomaterials in wound healing [124]. The rich biodiversity, advanced scientific research, and thriving healthcare industries of India, China, Japan, Korea, Singapore, Malaysia, Indonesia, Thailand, and many other Asian countries make this continent an ideal hub for the development and production of innovative biomaterials [125]. With a diverse array of natural resources and traditional medicine practices, Asia offers a vast pool of materials and knowledge that can be harnessed to create effective wound-healing solutions [126]. Furthermore, Asia’s rapidly growing economies and increasing investments in research and development provide a conducive environment for collaboration between academia, industry, and healthcare sectors, fostering the translation of cutting-edge research into practical applications [127]. As Asia continues to harness its unique strengths and capabilities, it is poised to play a pivotal role in meeting the growing global demand for biomaterials in wound healing, benefiting countless individuals worldwide.

Recent progress in healthcare management has extended worldwide life expectancy from 67.1 years in 2000 to 73.2 years in 2020 [128]. Furthermore, three of the top five countries ranked in life expectancy are of Asian demographic, namely Japan (2nd, 85.03 years), Macao (3rd, 84.68 years), and Singapore (5th, 84.07 years). According to WHO, by 2030, 1 in 6 people in the world will be aged ≥60 years. At this time, the share of the population aged ≥60 will increase from 1 billion in 2020 to 1.4 billion. By 2050, the world’s population of people ≥60 will double (2.1 billion). The number of persons aged ≥80 is expected to triple between 2020 and 2050 to reach 426 million. By 2050, two-thirds of the world’s population ≥60 will live in low- and middle-income countries [6]. Consequently, geriatric care associated to wounds will be a major industrial driver in Asia.

**Table 5:** Global market forecasts for biomaterials, 2020 to 2030 period.

Forecasting Period (Years)	CAGR (%)	Reference Value (USD Billions)	Forecasted Value (USD Billions)	Reference
2020–2027	12.2	110.0	245.6	[117]
2020–2027	15.2	109.4	390.9	[120]
2021–2030	12.7	65.0	212.4	[118]
2022–2030	15.4	135.4	488.7	[116]
2022–2030	12.2	121.4	343.7	[119]

### 5.2. Barriers in the Process to Meet the Growing Demand for Biomaterials

Unfortunately, the same barriers that have existed before, remain today [129,130]. Diminishing sources or depletion of raw materials are always a competitive issue in most industries. Hence, it often leads to more drastic acts or regulations, which ultimately serve to limit or prohibit new players to access the market, and this is in turn translated into higher efforts and costs for the new industries and, possibly, and impediment for the smaller ones to partake. As is the case of all markets that are in similar conditions, as smaller companies merge or are acquired by larger entities, the market becomes less diverse and discouraged from changing. This, in turn, creates a monopolizing effect between pre-existing or established conglomerates. Downstream, consumers are constantly threatened by price escalation, regardless of life-threatening situations.

In contrast to the strengths of the USA and Europe mentioned in Section 5.1, there are several countries with an unexplored potential for the biomaterial industry. This lack of knowledge is primarily due to insufficient financial, labor, and raw material resources, as well as inadequate technology infrastructure and customer/market relations, among other factors. Additionally, a considerable number of countries lack the necessary governing bodies or guidelines to facilitate the development of this industry. Recognizing the significant benefits that the biomaterial industry has contributed to the medical, economic, and educational sectors, Asian countries have started exploring the production of biomaterial products. By adopting regulatory bodies or guidelines similar to those in the Western countries, it is possible to prevent any false or malicious entities from exploiting the market [131,132]. As a result, Asia was recently cited as the fastest growing region [116,117,118,119,120]. Most of these countries include financial powerhouses such as India, China, Japan, Korea, Singapore, and specific countries in the Middle East, while others that follow closely include Malaysia, Indonesia, and Thailand. This is important because Asia remains an untapped potential and their willingness to explore further into this industry is a good sign. It is possible that a minimally biased and diversified market could exists with greater emphasis on Research and Development of new or improved versions of existing products. This model could also test more resource-efficient and cost-saving methods, relative to the challenges plaguing the West. 

The success of biomaterial products is also due to the prevalence of various health problems. While the US has led the way in impairments associated with metabolism such as obesity and diabetes for years, an increase in new cases has now been reported in Asia [133,134,135,136]. These diseases impair the innate recovery or the natural homeostasis of those affected. Therefore, a reliance on extrinsic methods through surgical interventions, drugs or medical devices is necessary. However, the treatability of these conditions should not be exploited for benefit purposes but used only when necessary and to raise medical awareness.

## 6. Global Regulatory Issues for Wound-Healing Products

Adherence to quality standards is crucial for the safe use of a medical device; otherwise, it can result in several clinical cases, such as the one we have observed with a medical device causing visual impairment in multiple countries [137,138].

### 6.1. USA Regulatory Issues

The Food and Drug Administration (FDA) oversees the regulatory framework for wound-healing products in the United States [139], as the regulatory issues are an integral aspect of the healthcare business, especially for wound-healing treatments. The FDA plays a crucial role in ensuring that wound-healing products are safe, effective, and in accordance with applicable rules. In the USA, the regulation procedure for wound-healing products can be difficult and time-consuming. Wound-healing products are classified as medical devices and are subject to the FDA’s Center for Devices and Radiological Health’s regulatory standards (CDRH) [140]. Generally speaking, the regulatory procedure for wound-healing devices consists of two phases: premarket review and postmarked monitoring [141]. Prior to allowing a wound-healing product to be commercialized in the United States, the FDA assesses it through the premarket review procedure. The premarket review procedure consists of several steps, including product categorization, device testing, and the filing of a premarket notice or premarket approval application.

Classification of a product is a crucial stage in the premarket evaluation process [142,143]. Class I devices are deemed low-risk, and are only subject to general regulations, such as labelling requirements and good manufacturing procedures. Class II medical devices are moderately risky and are subject to stringent regulations, including performance criteria and postmarket surveillance. Class III devices are regarded as high-risk and require clearance prior to commercial release. Testing of the device is another crucial phase in the premarket evaluation procedure. The FDA demands testing of wound-healing products to guarantee their safety and effectiveness. The testing standards differ depending on the classification of the device. Class I medical devices are normally exempt from testing, but Class II and Class III medical devices may require bench testing, animal testing, or clinical trials [144]. After classifying and testing a wound-healing product, the company must file an FDA premarket notice or premarket approval application. Class I and Class II devices must submit a premarket notification, whereas Class III ones must submit an application for premarket clearance [143]. The premarket notice or premarket approval application must contain information on the product, its intended use, its performance characteristics, and any testing or clinical studies completed.

Postmarket monitoring is the method through which the FDA monitors wound-healing products after they have been introduced to the marketplace [140,141]. Postmarket surveillance is to detect and address any safety or efficacy concerns that may develop after a medical device has been licensed for sale. Manufacturers must report adverse occurrences related with their devices to the FDA, and the FDA may conduct inspections or investigations to ensure that devices continue to comply with regulatory standards. In addition to the FDA, additional regulatory organizations may be engaged in wound-healing product regulation. For instance, the Federal Trade Commission (FTC) controls wound-healing product makers’ advertising and marketing claims. The Occupational Safety and Health Administration (OSHA) may regulate the workplace usage of wound-healing products [145].

Thus, regulatory issues are an integral part of the United States wound-healing product sector. The regulatory procedure consists of many processes, including product categorization, device testing, and premarket evaluation. The FDA plays a crucial role in ensuring that wound-healing products are safe, effective, and in accordance with applicable rules. Moreover, manufacturers must be aware of various regulatory agencies that may be engaged in wound-healing product regulation. Overall, the regulatory process can be difficult and time-consuming, but it is vital to guarantee that patients receive high-quality, safe, and effective wound-healing treatments.

### 6.2. European Regulatory Issues

The European Pharmacopoeia (Ph. Eur.) serves as the primary source of official quality standards for medicines and their ingredients within Europe. However, medical devices, which include products or equipment intended for medical purposes, are not categorized as medicines and, in accordance with the ‘classification rules’ set out in Annex VIII of Regulation (EU) 2017/745 on medical devices (MDR), a wound-healing product is classified according to Rule 4 as “Device that meet injured skin or mucous membrane”. These products are labelled as class I if they are used as simple wound dressings for skin or mucous membranes, as class IIa if used as dressings for wounds or injuries (such as nonmedicated, hydrogel dressings), and as class IIb if used for severe wounds on the skin or mucous membranes (e.g., ulcers, burns, severe decubitus wounds) or as a temporary skin substitute [146]. However, any wound-healing product incorporating, as an integral part, a substance which, if used separately, can be a medicinal product, as defined in point 2 of Article 1 of Directive 2001/83/EC, and that has an action ancillary to that of the device, are labelled as class III, in accordance with Rule 14. Therefore, a wound-healing dressing incorporating, for example, an antimicrobial agent which has an ancillary action on the wound, will be labelled as class III product. This classification necessitates the requirement of clinical studies to obtain a CE mark. Regardless of the classification of a wound-healing product, it is essential for all products to comply with the relevant obligations outlined in the MDR. However, the specific requirements vary based on the classification of the wound-healing product. A real case is represented by silk fibroin, a 100% biocompatible natural protein, totally free of any dangerous particle/molecule, with intrinsic antioxidant and reparative properties [146,147,148]. A clear candidate for natural polymer-based class I wound-care products. Silk fibroin could be a suitable natural polymer to produce high-performance wound caring product, if combined with antioxidant treatments, inflammation suppressors, and promotors of skin cells regeneration, e.g., silk protein hydrogels incorporating extracts of stem cells, medicinal plants, or stem cell secretome [149]. Nevertheless, since this silk fibroin incorporates substances with medicinal properties, when used independently, to enhance its therapeutic properties, its classification would pass from class I to class III.

A wound-healing product must adhere to the general safety and performance requirements, which include providing the necessary information, as specified in Annex I of the MDR, by the manufacturer. Additionally, the product should comply with reporting requirements outlined in the medical device vigilance system, obtain CE marking (with exceptions for custom-made devices and devices intended for clinical investigation, which must comply with the provisions of Art. 52.8 and Annex XIII or Articles 62–80, 82 and Annex XV. respectively), be assigned a Unique Device Identifier (UDI) number, and be registered in the electronic system in accordance with Article 29 of the MDR. 

### 6.3. Asian Regulatory Issues

The collective contributions of China, India, Japan, and South Korea demonstrate their crucial role in the wound-healing product market. The regulatory issues in these countries also play a vital role in ensuring the safety, efficacy, and quality of these products within their respective markets. Of particular interest because of its diversity and large population is Southeast Asia: Indonesia, Malaysia, the Philippines, Singapore, Thailand, Vietnam, Brunei, Cambodia, Laos, Burma, and Timor-Leste. To market the goods across this area, makers of wound-healing treatments must traverse several regulatory systems, as each nation has its own regulatory framework. In Asia also, wound-healing items are often classified as medical devices and are subject to special regulatory regimes [150]. Each nation in the area has its own regulatory authority responsible for assessing and approving medical devices, such as wound-healing treatments, prior to their commercialization and distribution inside its boundaries [151]. These regulatory authorities include the National Medical Products Administration (NMPA) in China, the Central Drugs Standard Control Organization (CDSCO) in India, the Pharmaceuticals and Medical Devices Agency (PMDA) in Japan, the Ministry of Food and Drug Safety (MFDS) in South Korea, the National Pharmaceutical Regulatory Agency (NPRA) in Malaysia, the National Agency for Drug and Food Control (NADFC) in Indonesia, the Health Sciences Authority (HSA) in Singapore and the Food and Drug Administration (FDA) in both Thailand and Philippines [152].

In Asia, the regulatory standards for wound-healing products differ by nation. However, product classification, clinical data, quality control, labeling, and packaging are examples of common needs [153]. The regulatory framework for wound-healing products is based on the norms and regulations established by each country’s regulatory organizations. The regulatory structure is intended to guarantee that all medical goods, including wound-healing treatments, are safe, effective, and of high quality. The essential components of the regulatory framework for wound-healing goods in Asia are as follows [75]:

- Product Classification: Medical products are categorized according to their intended purpose, level of risk, and mechanism of action. Depending on its mechanism of action and intended usage, wound-healing products are often classed as Class III or IV medical devices. However, the classification system ranges from Class I (low risk) to Class IV (high-risk). Each class has specific regulatory requirements and evaluation processes.
- Premarket Approval: Before a wound-healing product may be sold in Asia, it must be authorized by each country’s regulatory agency. The approval procedure requires the submission of a dossier covering all pertinent information regarding the product, such as its safety, effectiveness, and quality.
  ○ Clinical Trials and Evaluation: Depending on the risk classification, wound-healing products may be subject to clinical trials and evaluation to assess their safety and efficacy. Clinical data and evidence are required to demonstrate the product’s performance and benefits in promoting wound healing.
  ○ Quality Management System: Compliance with quality management system requirements, such as Good Manufacturing Practice (GMP) and ISO certification, is necessary for wound-healing product manufacturers. These standards ensure consistent product quality and safety throughout the manufacturing process. Harmonization with international regulations and standards with global guidelines is necessary to facilitate international trade and ensure product quality and safety.
- Postmarket Surveillance: Once a wound-healing product has been approved and sold, the regulatory authority performs postmarket monitoring to ensure that the product continues to fulfill safety, effectiveness, and quality criteria. Adverse event reporting, postmarket studies, and periodic safety updates are required to identify and address any potential safety concerns.
- Labeling and Advertising: The labeling and advertising of wound-healing products must adhere to the norms and guidelines established by each country’s regulatory agency. The labeling and advertising must be precise, honest, and not deceptive.

Thus, Asia’s regulatory environment for wound-healing products is complicated and demanding. Manufacturers must traverse different regulatory systems, show the safety and efficacy of their goods, and adhere to each country’s unique standards. Inadequate regulatory agencies’ coordination and inadequate resources can also result in manufacturing delays and cost increases. Notwithstanding these obstacles, Asia remains a very attractive market for wound-healing products, and firms that can effectively negotiate the regulatory framework will benefit from the expanding demand of this region.

## 7. Discussion and Conclusions

The use of natural compounds, stem cells, and biopolymers based on microRNAs (miRNAs) represents a paradigm shift in wound healing. Natural compounds, derived from plants or other biological sources, often possess anti-inflammatory and antimicrobial properties, promoting a favorable environment for healing. Stem cells, with their regenerative potential, can differentiate into various cell types, aiding tissue repair. Biopolymers, including miRNA-based ones, provide a scaffold for cell growth and deliver therapeutic molecules, facilitating the healing process.

The advantages of these innovative approaches include: (1) enhanced efficacy because natural compounds, stem cells, and miRNA-based biopolymers can work synergistically, addressing multiple aspects of wound healing simultaneously and potentially accelerating the process, (2) reduced side effects: compared to some conventional treatments, these natural approaches may have fewer side effects, making them more tolerable for patients, (3) precision in healing: stem cells and miRNA-based biopolymers offer a more targeted and precise approach to tissue regeneration, contributing to better overall wound-healing outcomes, (4) minimized scarring: the use of advanced biopolymers may contribute to minimizing scar formation during the remodeling phase, improving cosmetic results.

In conclusion, the global prevalence of wounds, especially chronic wounds, can be likened to a silent epidemic, disproportionately affecting populations worldwide, with more pronounced impacts in developing countries where early diagnosis is hindered by limited healthcare facilities. The intricate process of wound healing necessitates the utilization of specific care products. Additionally, with the world’s aging population on the rise and an escalation in diabetes and obesity cases among younger generations, there is a substantial surge in individuals susceptible to injuries, intensifying the demand for effective wound-care products. While this escalating demand poses a considerable financial burden, it simultaneously creates substantial opportunities for growth in the global wound-care products market. These products, primarily composed of natural polymeric materials, stem cells, and miRNA, showcase advancements, yet the complete healing of chronic wounds remains a challenging frontier, offering simultaneous challenges and significant market prospects.

For future investigations, exploring innovative approaches to enhance the efficacy of wound-care products, particularly in the context of chronic wounds, could be a promising avenue. Research focusing on personalized wound care strategies, integration of cutting-edge technologies, and a deeper understanding of the molecular mechanisms underlying chronic wound healing could provide valuable insights. Additionally, investigating cost-effective and accessible solutions for wound care, especially in resource-limited settings, would contribute to addressing global healthcare disparities.

## Figures and Tables

**Table 1:** Characteristics of selected biopolymers extensively used in wound management and their benefits in the healing process.

Biopolymer	Components	Sources	Benefits	Limitations	Study	Reference
Alginate	β-D-mannuronic acid and α-ʟ-guluronic acid linked by α-1,4 glycosidic linkages	Brown algae	Promote wound healing by activating macrophages to produce cytokines. High absorbance	High viscosity, nonhomogeneous, and nontransparent formulations	in vitro in vivo	[26]
Carboxymethyl cellulose	β-D-Glucose linked by β-1,4-glycosidic linkage	Modified from wood and cotton	Exudate absorbing capacity Retain the moisture	Weak antibacterial and antimicrobial properties, low mechanical strength	in vitro in vivo	[27]
Chitosan	*N*-acetyl glucosamine linked by β-1,4 glycosidic linkages	Shrimp and crabs	Antimicrobial, antibacterial, analgesic, hemostatic. Promotes neovascularization and dermis regeneration	Limited ability to certain antibacterial	in vitro in vivo	[28]
Collagen	Amino acid linked by amide linkage	Goat and ovine (sheep)	Enrichment of new collagen deposition Hemostatic ability. Control of proteolytic activity	Collagen of porcine and bovine sources. Risk of transmitting diseases, e.g., bovine spongiform encephalopathy (BSE), caused by prions	in vitro in vivo	[29]
Hyaluronic acid	D-glucuronic acid and *N*-acetyl-D-glu-cosamine linked by β-1,4 and β-1,3 glycosidic linkages	Bovine vitreous humor	Exudate absorption capacity. Anti-inflammatory. Induce cell adhesion	Weak mechanical properties, poor adhesion, and rapid degradation	in vitro in vivo ex vivo	[30,31]

**Table 2:** Few commercialized biopolymer-based wound-healing products in the market.

Products	Components	Benefits	Limitations	Company	Reference
ACTICOAT	Dressings with nanocrystalline silver technology	Sustained silver release into the wound exudate to help promote and retain a moist environment, when used with an appropriate secondary dressing. Bactericide.	Antimicrobial properties but helps the healing/healing process. It only prevents infections.	Smith & Nephew (UK)	[44]
Actisorb™	Activated carbon bandage with silver.	Activated carbon traps odor in the dressing and traps bacteria and toxins that impair the healing process. Bactericide.	Limited regenerative effects.	3M (USA)	[45]
Algisite M Calcium Alginate Dressing	Alginate Calcium	Fast gelling, high mannuronic acid fibers. Low fiber shed construction; it conforms to wound contours. It moisturizes wound environment, highly absorbent, biodegradable.	Calcium divalent cations can be released and exchange with monovalent cations in the surrounding media and dissolve the alginate gel.	Smith & Nephew (UK)	[38]
AmnioExcel^®^	Human placental amniotic fluid membrane.	Wound protection provided extracellular matrix proteins, growth factors and cytokines, which provides structural tissue and an environment for soft tissue reconstruction and regeneration.	Compositional differences between batches from different donors. Moderate effects, especially in chronic wounds.	Derma Sciences, Inc (USA)	[46]
AQUACEL^®^/Hydrofiber^®^	Sodium carboxymethyl-cellulose and regenerated cellulose fiber.	Adaptable and highly absorbent. In contact with the exudate, it creates a soft gel, maintaining a moist environment that facilitates progress in the healing process and autolytic debridement. It can be inserted into cavities or place on superficial lesions.	Bacteriostatic only. Healing improvement. Limited to simple and small wounds.	ConvaTec Group Plc (UK)	[47]
BIATAIN	Foam dressing with delicate silicone adhesive	Exudate absorption, even under compression. The 3D foam structure absorbs exudate, maintaining a moist environment.	Moderate effects especially on chronic and complex wounds.	Coloplast A/S (Denmark)	[48]
CalciCare™	Alginate, Calcium and Guluronic Acid and Silver Bandage	Absorbent. Hemostatic properties may assist in supporting the control of minor bleeding in superficial wounds. It helps maintaining a moist environment. Aids autolytic debridement.	Moderate effects especially on chronic and complex wounds.	Hollister Incorporated (USA)	[49]
Cutimed^®^ Epiona featuring 3D Matrix	Collagen and calcium alginate structure bandage	It can be easily molded to the surface of the wound. It does not contain chemical crosslinkers.	Recreates new extracellular matrix for regeneration: No regenerative cells or factors. Modest results.	Bsn Medical GmbH (Germany)	[50]
FD3101 (Wound Dressing)	Polyurethane foam and silver	High absorbency. Protection against water and bacteria. Nonadherent to the wound, no pain while removing the dressing.	Bacteriostatic only. It does not regenerate chronic skin wounds.	Triage Meditech Pvt (India)	[51]
Fibracol Plus Collagen Wound Dressing with Alginate	90% collagen. 10% calcium alginate	Can be cut to fit any size wound. Nonadherent and easily removable. Biodegradable. Low immunogenicity, noncarcinogenic, collagen synthesis and reepithelization, promotes cell proliferation, provides support for cell attachments. During granulation and at the beginning of the epithelization, it supports collagen fibrils and fibers’ formation.	Ca^2+^ divalent cations can be released and exchange with other monovalent cations in the surrounding media, resulting in the dissolving of the alginate gel	Johnson & Johnson (USA)	[43]
Granulox^®^	Purified hemoglobin	Highly purified hemoglobin takes oxygen molecules from the environment. The hemoglobin is distributed by the exudate of the wound and helps its healing.	Indicated for diabetic leg ulcers and venous ulcers. Moderate results.	Mölnlycke Health Care AB (Sweden)	[52]
HyperOil	Neem (Azadirachtin). Hypericum (Hyper forin).	Infection prevention, re-epithelialization, fibrinolytic activity, cleansing activity. Skin regeneration and elasticity promoter. For all kind of wounds (acute, chronic, infected). Biodegradable, nontoxic, noncarcinogenic. Both, gel, and oil formulation	Low antimicrobial properties.	RI.MOS. srl (Italy)	[42]
KALTOSTAT^®^	Calcium/sodium and alginate dressings	For ulcers and traumatic wounds. It improves healing. On contact with exudate, it forms a moist, firm, absorbent gel.	Moderate effects especially on deep and complex wounds	ConvaTec Group Plc (UK)	[53]
Kito Activator Chitosan Hydrogel Barrier	Chitosan Hydrogel	Synergy effect between kito activator and HR-chitosan depressing. Hemostatic, quick coagulation by strengthening ionic bonds with red blood cell and platelet. Antimicrobial, anti-inflammatory, deodorant. Nonpreservative, nonbinding, nonantibiotic. Biodegradable, nontoxic, noncarcinogenic.	Nothing found.	Endovision (Korea)	[40]
Medihoney HoneyColloid Dressing	Active leptospermum honey. Hydrocolloidal gelling agent	Helps reduce overall wound pH. Natural and safe. Effective in all wound-healing stages. High osmolarity helps cleansing-debriding. Moisturizing, biodegradable, nontoxic, noncarcinogenic.	May be increase level of exudate upon initial use. Only suitable for moderately exuding wounds.	Derma Sciences (USA)	[41]
Mepilex^®^ Ag	Foam bandage containing silver	Antibacterial, antifungal. Improves healing time. Atraumatic during dressing changes. Rapid and sustained activity.	Moderate effects, limited to certain types of wounds (e.g., burns)	Mölnlycke Health Care AB (Sweden)	[54]
NeutroPhase	Hypochlorous acid	Cleaning and debridement, from wounds and neutralization of toxins.	Bacteriostatic only. Not substantial speed up of chronic wounds healing.	Novabay Pharmaceutical, Inc. (USA)	[55]
Omnigraft	Bilayer matrix enriched in C6S collagen and silicone	Reduces inflammation, maintains moisture, and promotes cell and vascular growth in the wound.	Limited repairing effects, especially in chronic and complex wounds.	Derma Sciences, Inc (USA)	[56]
PriMatrix	Acellular dermal matrix enriched in type III collagen	Derived from fetal bovine dermis. It supports cell repopulation and revascularization critical in wound healing. Type III collagen helps tissue development and healing.	Limited repairing effects, especially in chronic and complex wounds.	Derma Sciences, Inc (USA)	[57]
Promogran Prisma^TM^	Collagen, oxidized regenerated cellulose (ORC) and silver-ORC bandage	In presence of exudate, it transforms into a soft biodegradable gel. It promotes granulation. It starts wounds that have been stalled in the inflammatory stage. Antimicrobial.	Moderate restorative effects	3M (USA)	[58]
REGRANEX	Recombinant platelet-derived growth factor	The only FDA-approved PDGF for diabetic neuropathic ulcers treatment. It increases tissue growth, re-epithelialization and revascularization rate.	Moderate cure rates in diabetic neuropathic ulcers	Smith & Nephew (UK)	[59]
Restore™	Hydrocolloid dressing	Occlusive dressing, impermeable to microorganisms, urine, and feces. With a disposable wound measuring guide. Heat-activated, self-adhesive inner layer maintains moist while absorbing wound exudates.	Barrier for bacterial and viral infections. Limited healing effects. Moderate exudate prevention.	Hollister Incorporated (USA)	[60]
SILVERCEL™	Alginate bandage, methylcellulose and silver	Antimicrobial barrier to reduce the risk of infection. Improves healing.	Limited effects on large and complex wounds.	Acelity L.P. Inc (USA)	[61]
Suprasorb C Collagen Wound Dressing	Collagen	Porous structure, absorbs fluids, debris and proinflammatory proteases and cytokines. It accelerates granulation tissue formation, induces fibroblasts migration and collagen synthesis. It supports proliferation and migration of epidermal cells. Biodegradable, nontoxic, noncarcinogenic.	Low machinal strength. Low antiseptic properties.	Lohmann & Rauscher (Germany)	[39]
V.A.C.^®^ Therapy	Programmable device	Negative compression therapy. Accelerates the healing process (reducing edema and promoting blood perfusion).	Very modest results; it prevents further worsening of wound. Very long-term treatments.	Acelity L.P. Inc (USA)	[62]
VTG2901	Programmable device for compression therapy (negative pressure).	Negative compression therapy. Accelerates the healing process (reducing edema and promoting blood perfusion). Removes excess fluid and reduces edema. Protects wound from microbes.	Very modest results, although it prevents further worsening of the wound. Very long-lasting treatment.	Triage Meditech Pvt (India)	[63]

**Table 3:** Wound-healing products based on animal origin stem cells under clinical trials.

Product	Components	Problem, Deficiencies, Limitation	Company	Reference
allo-APZ2	ABCB5- mesenchymal stem cells	Limitations inherent to the use of stem cells (contamination, allogeneic transplantation, modest healing)	Rheacell (Germany)	[80]
TruStem	Hematopoietic and mesenchymal stem cells	Limitations inherent to the use of stem cells (contamination, allogeneic transplantation, modest healing). Indefinite response time (weeks-to-months to notice therapeutic effects)	TruStem Cell Therapy (USA)	[82]
XSTEM	Human Stem Cells. Integrin α10β1	Inherent limitations of stem cell use (cell line contamination, allogeneic transplant compatibility, modest healing)	Xintela (Sweden)	[83]

## References

[1] Rodrigues M., Kosaric N., Bonham C.A., Gurtner G.C. (2019). Wound Healing: A Cellular Perspective. Physiol. Rev..

[2] Dhivya S., Padma V.V., Santhini E. (2015). Wound Dressings—A Review. BioMedicine.

[3] Rezvani Ghomi E., Khalili S., Nouri Khorasani S., Esmaeely Neisiany R., Ramakrishna S. (2019). Wound Dressings: Current Advances and Future Directions. J. Appl. Polym. Sci..

[4] Heyer K., Augustin M., Protz K., Herberger K., Spehr C., Rustenbach S.J. (2013). Effectiveness of Advanced versus Conventional Wound Dressings on Healing of Chronic Wounds: Systematic Review and Meta-Analysis. Dermatology.

[5] Denny K., Lawand C., Perry S. (2014). Compromised Wounds in Canada. Healthc. Q..

[6] World Health Organization (2022). Ageing and Health. World Health Organization. https://www.who.int/news-room/fact-sheets/detail/ageing-and-health#:~:text=At%20this%20time%20the%20share,2050%20to%20reach%20426%20million.

[7] Ageing Europe—Looking at the Lives of Older People in the EU—Eurostat 2019 Report. https://www.age-platform.eu/publications/ageing-europe-looking-lives-older-people-eu-eurostat-2019-report.

[8] Office for National Statistics Overview of the UK Population.

[9] Mark M., Paola S., Lillian K. Fact Sheet: Aging in the United States. https://www.prb.org/resources/fact-sheet-aging-in-the-united-states/.

[10] He W., Gookind D., Kowal P., Almasarweh W.I.S., Giang T.L., Islam M.M., Lee S., Teerawichitchainan B., Tey N.P. (2022). Asia Aging: Demographic, Economic, and Health Transitions.

[11] Malaysia Attained Ageing Nation Status. Department of Statistics Malaysia Official Portal. https://www.dosm.gov.my/v1/index.php?r=column/cthemeByCat&cat=155&bul_id=OVByWjg5YkQ3MWFZRTN5bDJiaEVhZz09&menu_id=L0pheU43NWJwRWVSZklWdzQ4TlhUUT09.

[12] Cross K., Harding K. (2022). Risk Profiling in the Prevention and Treatment of Chronic Wounds Using Artificial Intelligence. Int. Wound J..

[13] Guest J.F., Fuller G.W., Vowden P. (2020). Cohort Study Evaluating the Burden of Wounds to the UK’s National Health Service in 2017/2018: Update from 2012/2013. BMJ Open.

[14] Pokorná A., Leaper D. (2014). Assessment and Documentation of Non-healing, Chronic Wounds in Inpatient Health Care Facilities in the Czech Republic: An Evaluation Study. Int. Wound J..

[15] Pragadheeswaran M., Sankar lingam P., Balan Y., Pyati A.K. (2022). A Comparative Study Between Vacuum Dressing and Normal Saline Dressing for Chronic Non-Healing Ulcers. Cureus.

[16] LinkedIn Is Malaysian Market Ripe for Advanced Wound Care Products?. https://www.linkedin.com/pulse/malaysian-market-ripe-advanced-wound-care-products-ariyanchira/.

[17] Advanced Wound Care Market Size to Hit USD 45.33 Bn by 2032. https://www.precedenceresearch.com/advanced-wound-care-market.

[18] Advanced Wound Care Market Share, Growth. Forecast [2030]. https://www.fortunebusinessinsights.com/industry-reports/advanced-wound-care-market-100060.

[19] Sen C.K. (2019). Human Wounds and Its Burden: An Updated Compendium of Estimates. Adv. Wound Care.

[20] South East Asia Market Study on Disposable Medical Supplies: Cardiovascular Procedures Account for Over a Quarter of Overall Demand. https://www.persistencemarketresearch.com/market-research/south-east-asia-disposable-medical-supplies-market.asp.

[21] Freedman B.R., Hwang C., Talbot S., Hibler B., Matoori S., Mooney D.J. (2023). Breakthrough treatments for accelerated wound healing. Sci. Adv..

[22] Wound Healing Market. https://www.transparencymarketresearch.com/wound-healing-market.html.

[23] Sahana T.G., Rekha P.D. (2018). Biopolymers: Applications in Wound Healing and Skin Tissue Engineering. Mol. Biol. Rep..

[24] Neha R., Radha R., Rakesh P., Madhu G. (2022). Biopolymers and Treatment Strategies for Wound Healing: An Insight View. Int. J. Polym. Mater. Polym. Biomater..

[25] Gardikiotis I., Cojocaru F.-D., Mihai C.-T., Balan V., Dodi G. (2022). Borrowing the Features of Biopolymers for Emerging Wound Healing Dressings: A Review. Int. J. Mol. Sci..

[26] Postolović K., Ljujić B., Kovačević M.M., Đorđević S., Nikolić S., Živanović S., Stanić Z. (2022). Optimization, Characterization, and Evaluation of Carrageenan/Alginate/Poloxamer/Curcumin Hydrogel Film as a Functional Wound Dressing Material. Mater. Today Commun..

[27] Diaz-Gomez L., Gonzalez-Prada I., Millan R., Da Silva-Candal A., Bugallo-Casal A., Campos F., Concheiro A., Alvarez-Lorenzo C. (2022). 3D Printed Carboxymethyl Cellulose Scaffolds for Autologous Growth Factors Delivery in Wound Healing. Carbohydr. Polym..

[28] Hou L., Wang W., Wang M.-K., Song X.-S. (2022). Acceleration of Healing in Full-Thickness Wound by Chitosan-Binding BFGF and Antimicrobial Peptide Modification Chitosan Membrane. Front. Bioeng. Biotechnol..

[29] Mh Busra F., Rajab N.F., Tabata Y., Saim A.B., Idrus R.B.H., Chowdhury S.R. (2019). Rapid Treatment of Full-Thickness Skin Loss Using Ovine Tendon Collagen Type I Scaffold with Skin Cells. J. Tissue Eng. Regen. Med..

[30] Hussain Z., Pandey M., Thu H.E., Kaur T., Jia G.W., Ying P.C., Xian T.M., Abourehab M.A.S. (2022). Hyaluronic Acid Functionalization Improves Dermal Targeting of Polymeric Nanoparticles for Management of Burn Wounds: In Vitro, Ex Vivo and in Vivo Evaluations. Biomed. Pharmacother..

[31] Zhang Y., Zheng Y., Shu F., Zhou R., Bao B., Xiao S., Li K., Lin Q., Zhu L., Xia Z. (2022). In Situ-Formed Adhesive Hyaluronic Acid Hydrogel with Prolonged Amnion-Derived Conditioned Medium Release for Diabetic Wound Repair. Carbohydr. Polym..

[32] Yang Y., Xu L., Wang J., Meng Q., Zhong S., Gao Y., Cui X. (2022). Recent Advances in Polysaccharide-Based Self-Healing Hydrogels for Biomedical Applications. Carbohydr. Polym..

[33] Böhm S., Strauß C., Stoiber S., Kasper C., Charwat V. (2017). Impact of Source and Manufacturing of Collagen Matrices on Fibroblast Cell Growth and Platelet Aggregation. Materials.

[34] Busra F.M., Lokanathan Y., Nadzir M.M., Saim A., Idrus R.B.H., Chowdhury S.R. (2017). Attachment, Proliferation, and Morphological Properties of Human Dermal Fibroblasts on Ovine Tendon Collagen Scaffolds: A Comparative Study. Malays. J. Med. Sci..

[35] Sultan M.T., Lee O.J., Kim S.H., Ju H.W., Park C.H. (2018). Silk Fibroin in Wound Healing Process. Adv. Exp. Med. Biol..

[36] Dong M., Mao Y., Zhao Z., Zhang J., Zhu L., Chen L., Cao L. (2022). Novel Fabrication of Antibiotic Containing Multifunctional Silk Fibroin Injectable Hydrogel Dressing to Enhance Bactericidal Action and Wound Healing Efficiency on Burn Wound: In Vitro and in Vivo Evaluations. Int. Wound J..

[37] Neamtu B., Barbu A., Negrea M.O., Berghea-Neamțu C.Ș., Popescu D., Zăhan M., Mireșan V. (2022). Carrageenan-Based Compounds as Wound Healing Materials. Int. J. Mol. Sci..

[38] Algisite M Calcium Alginate Dressing. Medical Dressings. https://www.smith-nephew.com/es-es/health-care-professionals/products/advanced-wound-management/algisite-ppl.

[39] Suprasorb C Collagen Wound Dressing. https://www.lohmann-rauscher.com/en/products/wound-care/modern-wound-care/suprasorb-c/.

[40] Kito Activator Chitosan Wound Healing Hydrogel Barrier. http://eng.endovision.co.kr/portfolio-item/kito-activator/.

[41] Medihoney HoneyColloid Leptospermum Hydrocolloid Dressings. https://www.products.integralife.com/outpatient-clinic-private-office/category/wound-reconstruction-care-outpatient-clinic-private-office.

[42] Hyperoil Hyperoil—For Any Wound, Any Time. https://hyperoil.com/.

[43] Fibracol Plus Collagen Wound Dressing with Alginate. https://www.vitalitymedical.com/johnson-and-johnson-fibracol-plus-collagen-wound-dressing-with-alginate.html.

[44] ACTICOAT https://www.smith-nephew.com/es-es/health-care-professionals/products/advanced-wound-management/acticoat-global#overview.

[45] 3M^TM^ Actisorb^TM^ Silver 220 Activated Charcoal Dressing with Silver. https://www.3m.co.za/3M/en_ZA/p/d/b5005265071/.

[46] AmnioExcel^®^ https://www.integralife.com/es/amnioexcel-amniotic-allograft-membrane/product/wound-reconstruction-care-outpatient-clinic-private-office-treat-amnioexcel-amniotic-allograft-membrane.

[47] AQUACEL^®^/Hydrofiber^®^. https://www.convatec.com/es-es/productos/cuidado-avanzados-de-heridas/tipo-de-herida/pc-wound-burns/aquacel-extra/.

[48] BIATAIN^®^ Silicone. https://productos.coloplast.com.ar/coloplast/heridas/biatain-silicone/biatain-silicone/.

[49] CalciCare^TM^ Calcium Alginate Dressing Calcium Alginate Dressings. Hollister US. https://www.hollister.com/en/products/wound-care-products/wound-dressings/calcium-alginate-dressings/calcicare-calcium-alginate-dressing#.

[50] Cutimed https://medical.essity.de/marken/cutimed.html.

[51] Triage Meditech Advanced Wound Dressings. https://www.triagemeditech.com/advance-wound-dressings.

[52] Granulox^®^ https://www.molnlycke.es/productos-soluciones/granulox/.

[53] Kaltostat^®^ https://www.convatec.com/es-es/productos/cuidado-avanzados-de-heridas/tipo-de-herida/pc-wound-diabetic-foot-ulcers/b68c3a76-bd08-404d-a982-efeb45b1879d/.

[54] Mepilex^®^ Ag. https://www.molnlycke.es/productos-soluciones/mepilex-ag/.

[55] NeutroPhase https://novabay.com/products/neutrophase/.

[56] Omnigraft https://www.integralife.com/es/omnigraft-dermal-regeneration-matrix/product/wound-reconstruction-care-outpatient-clinic-private-office-treat-omnigraft-dermal-regeneration-matrix.

[57] PriMatrix^®^ Dermal Repair Scaffold. https://www.integralife.com/primatrix-dermal-repair-scaffold/product/wound-reconstruction-care-inpatient-acute-or-primatrix-dermal-repair-scaffold.

[58] 3M^TM^ Promogran Prisma^TM^ Collagen Matrix with ORC and Silver. https://www.3m.com/3M/en_US/p/d/b5005265080/.

[59] REGRANEX https://www.smith-nephew.com/en-us/health-care-professionals/products/advanced-wound-management/regranex#productfeatures.

[60] Hollister US Restore^TM^ Hydrocolloid Dressing with Foam Backing. Wound Dressings. https://www.hollister.com/en/products/wound-care-products/wound-dressings/hydrocolloid-dressings/restore-hydrocolloid-dressing-with-foam-backing.

[61] SILVERCEL^TM^. https://www.acelity.com/healthcare-professionals/global-product-catalog/catalog/silvercel-dressing.

[62] V.A.C.^®^ Therapy https://www.acelity.com/healthcare-professionals/global-product-catalog/catalog/vac-freedom.

[63] Insticare VTG2901 Manufacturer & Supplier Triage Meditech. https://www.triagemeditech.com/vtg-2901-new-advanced-solution.

[64] Ayavoo T., Murugesan K., Gnanasekaran A. (2021). Roles and Mechanisms of Stem Cell in Wound Healing. Stem Cell Investig..

[65] Srivastava G.K., Rodriguez-Crespo D., Fernandez-Bueno I., Pastor J.C. (2022). Factors Influencing Mesenchymal Stromal Cells in in Vitro Cellular Models to Study Retinal Pigment Epithelial Cell Rescue. Hum. Cell.

[66] Di Lauro S., Garcia-Gutierrez M.T., Fernandez-Bueno I. (2020). Quantification of Pigment Epithelium-Derived Factor (PEDF) in an Ex Vivo Coculture of Retinal Pigment Epithelium Cells and Neuroretina. J. Allbiosolution.

[67] Alonso-Alonso M.L., Srivastava G.K., Usategui-Martín R., García-Gutierrez M.T., Pastor J.C., Fernandez-Bueno I. (2020). Mesenchymal Stem Cell Secretome Enhancement by Nicotinamide and Vasoactive Intestinal Peptide: A New Therapeutic Approach for Retinal Degenerative Diseases. Stem Cells Int..

[68] Trzyna A., Banaś-Ząbczyk A. (2021). Adipose-Derived Stem Cells Secretome and Its Potential Application in “Stem Cell-Free Therapy”. Biomolecules.

[69] Gorecka J., Kostiuk V., Fereydooni A., Gonzalez L., Luo J., Dash B., Isaji T., Ono S., Liu S., Lee S.R. (2019). The Potential and Limitations of Induced Pluripotent Stem Cells to Achieve Wound Healing. Stem Cell Res. Ther..

[70] Paik D.T., Chandy M., Wu J.C. (2020). Patient and Disease–Specific Induced Pluripotent Stem Cells for Discovery of Personalized Cardiovascular Drugs and Therapeutics. Pharmacol. Rev..

[71] Nourian Dehkordi A., Mirahmadi Babaheydari F., Chehelgerdi M., Raeisi Dehkordi S. (2019). Skin Tissue Engineering: Wound Healing Based on Stem-Cell-Based Therapeutic Strategies. Stem Cell Res. Ther..

[72] Maarof M., Mohd Nadzir M., Sin Mun L., Fauzi M.B., Chowdhury S.R., Idrus R.B.H., Lokanathan Y. (2021). Hybrid Collagen Hydrogel/Chondroitin-4-Sulphate Fortified with Dermal Fibroblast Conditioned Medium for Skin Therapeutic Application. Polymers.

[73] Ling-Chun C., Shyr-Yi L., Ming-Thau S., Ching-Hua S., Hong-Liang L., Chien-Ming H. (2021). Fabrication and Characterization of Rhizochitosan and Its Incorporation with Platelet Concentrates to Promote Wound Healing. Carbohydr. Polym..

[74] Md Fadilah N.I., Mohd Abdul Kader Jailani M.S., Badrul Hisham M.A.I., Sunthar Raj N., Shamsuddin S.A., Ng M.H., Fauzi M.B., Maarof M. (2022). Cell Secretomes for Wound Healing and Tissue Regeneration: Next Generation Acellular Based Tissue Engineered Products. J. Tissue Eng..

[75] Catanzano O., Quaglia F., Boateng J.S. (2021). Wound Dressings as Growth Factor Delivery Platforms for Chronic Wound Healing. Expert Opin. Drug Deliv..

[76] Mashiko T., Takada H., Wu S.-H., Kanayama K., Feng J., Tashiro K., Asahi R., Sunaga A., Hoshi K., Kurisaki A. (2018). Therapeutic Effects of a Recombinant Human Collagen Peptide Bioscaffold with Human Adipose-Derived Stem Cells on Impaired Wound Healing after Radiotherapy. J. Tissue Eng. Regen. Med..

[77] Sezer A.D., Cevher E., Sezer A.D., Cevher E. (2011). Biopolymers as Wound Healing Materials: Challenges and New Strategies.

[78] Growth Factors in Wound Healing—A Review. https://biomedpharmajournal.org/vol14no3/growth-factors-in-wound-healing-a-review/.

[79] Aguiar Koga B.A., Fernandes L.A., Fratini P., Sogayar M.C., Carreira A.C.O. (2023). Role of MSC-derived Small Extracellular Vesicles in Tissue Repair and Regeneration. Front. Cell Dev. Biol..

[80] RHEACELL >< Technology 2023. https://www.rheacell.com/.

[81] (2019). Allo-APZ2. https://www.ema.europa.eu/en/medicines/human/orphan-designations/eu3192160.

[82] (2023). Home of TruStem Cell Therapy—Top U.S. Stem Cell Therapy & Treatment Center TruStem Cell Therapy^TM^. https://trustemcell.com/.

[83] (2022). Xintela XSTEM. https://www.xintela.se/en/press-release?slug=xintela-starts-clinical-study-of-xstem-r-in-knee-osteoarthritis.

[84] Avita Medical A Feasibility Study of the ReGenerCellTM Autologous Cell Harvesting Device for Diabetic Foot Ulcer. https://clinicaltrials.gov/study/NCT02799121.

[85] Kimmel H., Gittleman H. (2017). Retrospective Observational Analysis of the Use of an Architecturally Unique Dermal Regeneration Template (Derma Pure^®^) for the Treatment of Hard-to-Heal Wounds. Int. Wound J..

[86] Lavery L.A., Fulmer J., Shebetka K.A., Regulski M., Vayser D., Fried D., Kashefsky H., Owings T.M., Nadarajah J., Grafix Diabetic Foot Ulcer Study Group (2014). The Efficacy and Safety of Grafix^®^ for the Treatment of Chronic Diabetic Foot Ulcers: Results of a Multi-Centre, Controlled, Randomised, Blinded, Clinical Trial. Int. Wound J..

[87] Ruiz-Cañada C., Bernabé-García Á., Liarte S., Rodríguez-Valiente M., Nicolás F.J. (2021). Chronic Wound Healing by Amniotic Membrane: TGF-β and EGF Signaling Modulation in Re-Epithelialization. Front. Bioeng. Biotechnol..

[88] O’Brien J., Hayder H., Zayed Y., Peng C. (2018). Overview of MicroRNA Biogenesis, Mechanisms of Actions, and Circulation. Front. Endocrinol..

[89] Meng Z., Zhou D., Gao Y., Zeng M., Wang W. (2018). MiRNA Delivery for Skin Wound Healing. Adv. Drug Deliv. Rev..

[90] Weiss C.N., Ito K. (2017). A Macro View of MicroRNAs: The Discovery of MicroRNAs and Their Role in Hematopoiesis and Hematologic Disease. Int. Rev. Cell Mol. Biol..

[91] Banerjee J., Sen C.K. (2015). MicroRNA and Wound Healing. Adv. Exp. Med. Biol..

[92] Singh R., Shitiz K., Singh A. (2017). Chitin and Chitosan: Biopolymers for Wound Management. Int. Wound J..

[93] Ramamoorth M., Narvekar A. (2015). Non Viral Vectors in Gene Therapy—An Overview. J. Clin. Diagn. Res..

[94] Kolimi P., Narala S., Nyavanandi D., Youssef A.A.A., Dudhipala N. (2022). Innovative Treatment Strategies to Accelerate Wound Healing: Trajectory and Recent Advancements. Cells.

[95] Zeng X., Chen B., Wang L., Sun Y., Jin Z., Liu X., Ouyang L., Liao Y. (2023). Chitosan@Puerarin Hydrogel for Accelerated Wound Healing in Diabetic Subjects by MiR-29ab1 Mediated Inflammatory Axis Suppression. Bioact. Mater..

[96] Zhang W., Qu X., Zhu Z., Wang L., Qi Q., Zhou P., Wang X., Li W. (2021). Inhibition of MiR-139-5p by Topical JTXK Gel Promotes Healing of Staphylococcus Aureus-Infected Skin Wounds. Cells Dev..

[97] Liu Z., Zhang L., Toma M.A., Li D., Bian X., Pastar I., Tomic-Canic M., Sommar P., Xu Landén N. (2022). Integrative Small and Long RNA Omics Analysis of Human Healing and Nonhealing Wounds Discovers Cooperating MicroRNAs as Therapeutic Targets. eLife.

[98] Li H., Han X., Zuo K., Li L., Liu J., Yuan X., Shen Y., Shao M., Pang D., Chu Y. (2018). MiR-23b Promotes Cutaneous Wound Healing through Inhibition of the Inflammatory Responses by Targeting ASK1. Acta Biochim. Biophys. Sin..

[99] Bi Q., Liu J., Wang X., Sun F. (2020). Downregulation of MiR-27b Promotes Skin Wound Healing in a Rat Model of Scald Burn by Promoting Fibroblast Proliferation. Exp. Ther. Med..

[100] Wu J., Li X., Li D., Ren X., Li Y., Herter E.K., Qian M., Toma M.-A., Wintler A.-M., Sérézal I.G. (2020). MicroRNA-34 Family Enhances Wound Inflammation by Targeting LGR4. J. Investig. Dermatol..

[101] Bi X., Zhou L., Liu Y., Gu J., Mi Q.-S. (2022). MicroRNA-146a Deficiency Delays Wound Healing in Normal and Diabetic Mice. Adv. Wound Care.

[102] Liu L., Chen R., Jia Z., Li X., Tang Y., Zhao X., Zhang S., Luo L., Fang Z., Zhang Y. (2022). Downregulation of Hsa-MiR-203 in Peripheral Blood and Wound Margin Tissue by Negative Pressure Wound Therapy Contributes to Wound Healing of Diabetic Foot Ulcers. Microvasc. Res..

[103] De Kerckhove M., Tanaka K., Umehara T., Okamoto M., Kanematsu S., Hayashi H., Yano H., Nishiura S., Tooyama S., Matsubayashi Y. (2018). Targeting MiR-223 in Neutrophils Enhances the Clearance of *Staphylococcus aureus* in Infected Wounds. EMBO Mol. Med..

[104] Xie J., Wu W., Zheng L., Lin X., Tai Y., Wang Y., Wang L. (2022). Roles of MicroRNA-21 in Skin Wound Healing: A Comprehensive Review. Front. Pharmacol..

[105] Li D., Li X.I., Wang A., Meisgen F., Pivarcsi A., Sonkoly E., Ståhle M., Landén N.X. (2015). MicroRNA-31 Promotes Skin Wound Healing by Enhancing Keratinocyte Proliferation and Migration. J. Investig. Dermatol..

[106] Zhang X.-F., Wang T., Wang Z.-X., Huang K.-P., Zhang Y.-W., Wang G.-L., Zhang H.-J., Chen Z.-H., Wang C.-Y., Zhang J.-X. (2021). Hypoxic UcMSC-Secreted Exosomal MiR-125b Promotes Endothelial Cell Survival and Migration during Wound Healing by Targeting TP53INP1. Mol. Ther.—Nucleic Acids.

[107] Jiang B., Tang Y., Wang H., Chen C., Yu W., Sun H., Duan M., Lin X., Liang P. (2020). Down-Regulation of Long Non-Coding RNA HOTAIR Promotes Angiogenesis via Regulating MiR-126/SCEL Pathways in Burn Wound Healing. Cell Death Dis..

[108] Yang L., Zheng Z., Zhou Q., Bai X., Fan L., Yang C., Su L., Hu D. (2017). MiR-155 Promotes Cutaneous Wound Healing through Enhanced Keratinocytes Migration by MMP-2. J. Mol. Histol..

[109] Jin Y., Tymen S.D., Chen D., Fang Z.J., Zhao Y., Dragas D., Dai Y., Marucha P.T., Zhou X. (2013). MicroRNA-99 Family Targets AKT/MTOR Signaling Pathway in Dermal Wound Healing. PLoS ONE.

[110] Gallant-Behm C.L., Piper J., Lynch J.M., Seto A.G., Hong S.J., Mustoe T.A., Maari C., Pestano L.A., Dalby C.M., Jackson A.L. (2019). A MicroRNA-29 Mimic (Remlarsen) Represses Extracellular Matrix Expression and Fibroplasia in the Skin. J. Investig. Dermatol..

[111] Mulholland E.J., Dunne N., McCarthy H.O. (2017). MicroRNA as Therapeutic Targets for Chronic Wound Healing. Mol. Ther. Nucleic Acids.

[112] Hanna J., Hossain G.S., Kocerha J. (2019). The Potential for MicroRNA Therapeutics and Clinical Research. Front. Genet..

[113] Zomer H.D., Trentin A.G. (2018). Skin Wound Healing in Humans and Mice: Challenges in Translational Research. J. Dermatol. Sci..

[114] Chakraborty C., Sharma A.R., Sharma G., Lee S.-S. (2021). Therapeutic Advances of MiRNAs: A Preclinical and Clinical Update. J. Adv. Res..

[115] Hoversten K.P., Kiemele L.J., Stolp A.M., Takahashi P.Y., Verdoorn B.P. (2020). Prevention, Diagnosis, and Management of Chronic Wounds in Older Adults. Mayo Clin. Proc..

[116] (2021). Global Biomaterials Market Size & Share Report, 2022–2030. https://www.grandviewresearch.com/industry-analysis/biomaterials-industry.

[117] Biomaterials Market Size, Share and Industry Analysis, by Material (Metallic, Ceramic, Polymers, and Natural), by Application (Cardiovascular, Dental, Orthopedic, Plastic Surgery, Urology, Gastroenterology, and Others), and Regional Forecast, 2020–2027. Biomaterials Market Size, Growth | Global Industry Analysis, 2017. https://www.fortunebusinessinsights.com/biomaterials-market-102770.

[118] Allied Market Research Biomaterials Market Size: Key Analysis: Forecast—2030. https://www.alliedmarketresearch.com/biomaterials-market.

[119] Biomaterials Market (2022). Biomaterials Market Register a Revenue CAGR of 12.2%. https://www.reportsanddata.com/report-detail/biomaterials-market.

[120] Precedence Research Biomaterials Market Size, Share, and Growth Analysis Report by Product (Metallic, Ceramics, Natural, and Polymers) by Application (Ophthalmology, Cardiovascular, Dental, Wound Healing, Orthopedic, Plastic Surgery, Tissue Engineering, Neurology, and Others)—Global Industry Analysis, Trends, Regional Outlook and Forecasts 2020–2027. https://www.precedenceresearch.com/biomaterials-market#:~:text=As%20per%20Precedence%20Research%2C%20the,USD%20390.92%20billion%20by%202027.

[121] North America Biomaterials Market Analysis: 2022 to 2027. (2022). Market Data Forecast. https://www.marketdataforecast.com/market-reports/north-america-biomaterials-market.

[122] EMA (2018). Medical Devices. European Medicine Agency. https://www.ema.europa.eu/en/human-regulatory/overview/medical-devices.

[123] Fergal D. (2015). European Perspectives on Biomaterials for Health. Eur. Wound Manag. Assoc. J..

[124] APAC Biomaterials Market. http://www.marketdataforecast.com/.

[125] Rômulo RN A., Ierecê ML R. (2007). Biodiversity, Traditional Medicine and Public Health: Where Do They Meet?. J. Ethnobiol. Ethnomed..

[126] Shedoeva A., Leavesley D., Upton Z., Fan C. (2019). Wound Healing and the Use of Medicinal Plants. Evid.-Based Complement. Altern. Med..

[127] ASEAN (2021). ASEAN Investment Report 2020–2021.

[128] Worldometer Life expectancy of the World Population. https://www.worldometers.info/demographics/life-expectancy/.

[129] Uludağ H. (2014). Grand Challenges in Biomaterials. Front. Bioeng. Biotechnol..

[130] Williams D.F. (2019). Challenges with the Development of Biomaterials for Sustainable Tissue Engineering. Front. Bioeng. Biotechnol..

[131] Ebrahimi M., Mozafari M. (2020). Chapter 12—Standardization and Regulation of Biomaterials. Handbook of Biomaterials Biocompatibility.

[132] Ma C., Kuzma M.L., Bai X., Yang J. (2019). Biomaterial-Based Metabolic Regulation in Regenerative Engineering. Adv. Sci..

[133] CDC Overweight & Obesity. https://www.cdc.gov/obesity/index.html.

[134] Diabetes Quick Facts | Basics | Diabetes | CDC. https://www.cdc.gov/diabetes/basics/quick-facts.html.

[135] Zainuddin L.R.M., Isa N., Muda W.M.W., Mohamed H.J. (2011). The Prevalence of Metabolic Syndrome According to Various Definitions and Hypertriglyceridemic-Waist in Malaysian Adults. Int. J. Prev. Med..

[136] Ranasinghe P., Mathangasinghe Y., Jayawardena R., Hills A.P., Misra A. (2017). Prevalence and Trends of Metabolic Syndrome among Adults in the Asia-Pacific Region: A Systematic Review. BMC Public Health.

[137] Januschowski K., Irigoyen C., Pastor J.C., Srivastava G.K., Romano M.R., Heimann H., Stalmans P., Van Keer K., Boden K., Szurman P. (2018). Retinal Toxicity of Medical Devices Used during Vitreoretinal Surgery: A Critical Overview. Ophthalmologica.

[138] Kalaiselvan V., Shubhang A., Rajeev Singh R. (2023). A Systematic Review on Research Documentation of Ocular Medical Device Focused on Perfluorocarbon Liquid (PFCL). J. Allbiosolution.

[139] Iglesias-Lopez C., Agustí A., Obach M., Vallano A. (2019). Regulatory Framework for Advanced Therapy Medicinal Products in Europe and United States. Front. Pharmacol..

[140] Jarow J.P., Baxley J.H. (2015). Medical Devices: US Medical Device Regulation. Urol. Oncol..

[141] Sweet B.V., Schwemm A.K., Parsons D.M. (2011). Review of the Processes for FDA Oversight of Drugs, Medical Devices, and Combination Products. J. Manag. Care Pharm. JMCP.

[142] Bliznakov Z., Mitalas G., Pallikarakis N., Magjarevic R., Nagel J.H. (2007). Analysis and Classification of Medical Device Recalls. Proceedings of the World Congress on Medical Physics and Biomedical Engineering 2006.

[143] Oberweis C.V., Marchal J.A., López-Ruiz E., Gálvez-Martín P. (2020). A Worldwide Overview of Regulatory Frameworks for Tissue-Based Products. Tissue Eng. Part B. Rev..

[144] Kramer D.B., Xu S., Kesselheim A.S. (2012). Regulation of Medical Devices in the United States and European Union. N. Engl. J. Med..

[145] Lisa M.S., Steven W. (2019). Medical Marketing in the United States, 1997–2016. JAMA.

[146] Radley-Gardner O., Beale H., Zimmerman R. (2016). Fundamental Texts on European Private Law.

[147] Yonesi M., Garcia-Nieto M., Guinea G.V., Panetsos F., Pérez-Rigueiro J., González-Nieto D. (2021). Silk Fibroin: An Ancient Material for Repairing the Injured Nervous System. Pharmaceutics.

[148] Yonesi M., Ramos M., Ramirez-Castillejo C., Fernández-Serra R., Panetsos F., Belarra A., Chevalier M., Rojo F.J., Pérez-Rigueiro J., Guinea G.V. (2023). Resistance to Degradation of Silk Fibroin Hydrogels Exposed to Neuroinflammatory Environments. Polymers.

[149] Martín-Martín Y., Fernández-García L., Sanchez-Rebato M.H., Marí-Buyé N., Rojo F.J., Pérez-Rigueiro J., Ramos M., Guinea G.V., Panetsos F., González-Nieto D. (2019). Evaluation of Neurosecretome from Mesenchymal Stem Cells Encapsulated in Silk Fibroin Hydrogels. Sci. Rep..

[150] Jakovljevic M., Wu W., Merrick J., Cerda A., Varjacic M., Sugahara T. (2021). Asian Innovation in Pharmaceutical and Medical Device Industry—Beyond Tomorrow. J. Med. Econ..

[151] George B. (2010). Registration of Medical Devices. Perspect. Clin. Res..

[152] Mohit, Deep A., Khurana G., Kumar J., Monga A. (2019). Comparison of Regulatory Requirements for Registration of Pharmaceutical Drugs in Asean and GCC Regions. Appl. Clin. Res. Clin. Trials Regul. Aff..

[153] Aprile P., Letourneur D., Simon-Yarza T. (2020). Membranes for Guided Bone Regeneration: A Road from Bench to Bedside. Adv. Healthc. Mater..

